# Supplementary material for: Induced pluripotent stem cells of endangered avian species
Source: Commun Biol. 2022 Oct 24;5:1049. doi: 10.1038/s42003-022-03964-y (PMC9592614; doi:10.1038/s42003-022-03964-y)
Supplement: Supplementary file 2 — Supplementary Information [file 42003_2022_3964_MOESM2_ESM.pdf]

Supplementary information

Induced pluripotent stem cells of endangered avian species

Masafumi Katayama, Tomokazu Fukuda, Takehito Kaneko, Yuki Nakagawa, Atsushi Tajima, Mitsuru Naito, Hitomi Ohmaki, Daiji Endo, Makoto Asano, Takashi Nagamine, Yumiko Nakaya, Keisuke Saito, Yukiko Watanabe, Tetsuya Tani, Miho Inoue-Murayama, Nobuyoshi Nakajima, Manabu Onuma .

Corresponding authors:
Masafumi Katayama Ph.D.,
katayama.masafumi@nies.go.jp

Tomokazu Fukuda, Ph.D.,
tomofukuda009@gmail.com

Manabu Onuma DVM., Ph.D.,
E-mail: monuma@nies.go.jp

Supplemental methods

Generation of iPSCs with PB-R6F reprogramming vector.

PB-R6F reprogramming vectors were transfected into mouse, chicken, Okinawa rail, Japanese ptarmigan, and Blakiston's fish owl-derived fibroblasts using Lipofectamine 2000 transduction reagent (11668019; Thermo Fisher Scientific, Waltham, MA, USA). The PB-R6F reprogramming vector has been described in detail in our previous reports [1, 2]. After hygromycin selection (Wako Pure Chemical Industries, Osaka, Japan), transduced cells were reseeded onto a mouse embryonic fibroblast (MEF) feeder layer. Primary iPS cell-like colonies were selected and seeded onto new MEF feeder cell plates.

Okinawa rail, Japanese ptarmigan, and Blakiston's fish owl genome and amino acids.

Nucleic acid sequences derived from Okinawa rail, Japanese ptarmigan, and Blakiston's fish owl DNA were obtained using a next-generation sequencer (HiSeq and IonPGM). To obtain target genes (e.g., *Pou5*, *Sox2*, and *Nanog*), we searched all Okinawa rail, Japanese ptarmigan, and Blakiston's fish owl sequences using Blastn, with chicken mRNA sequences as queries. The sequences obtained were converted into amino acids, and a comparison of mouse, chicken, Okinawa rail, Japanese ptarmigan, and Blakiston's fish owl was performed using the T-Coffee website (http://tcoffee.vital - it.ch/apps/ tcoffee/index.html).

Comparison of characteristics of iPSCs with PB-R6F and PB-TAD-7F.

In addition to the PB-TAD-7F reprogramming vector, we used the PB-R6F vector (*M3O*, *Sox2*, *Klf4*, *c-Myc*, *Nanog*, and *Lin28*). There were no observable differences between iPSCs with PB-TAD-7F or PB-R6F in chicken and mouse iPSCs concerning morphology, pluripotency marker staining, and pluripotency-related gene expression (Supplementary Fig. 2 to 4). However, we observed differences between reprogramming vectors in Blakiston's fish owl-derived cells. In brief, the PB-TAD-7F reprogramming vector generated Blakiston's fish owl iPSCs, whereas the PB-R6F reprogramming vector was unable to generate Blakiston's fish owl iPSCs (Supplementary Fig. 2). Therefore, we conclude that the PB-TAD-7F reprogramming vector is more advantageous than the PB-R6F reprogramming vector for generating avian iPSCs. We analyzed the characteristics of iPSCs using the PB-TAD-7F reprogramming vector.

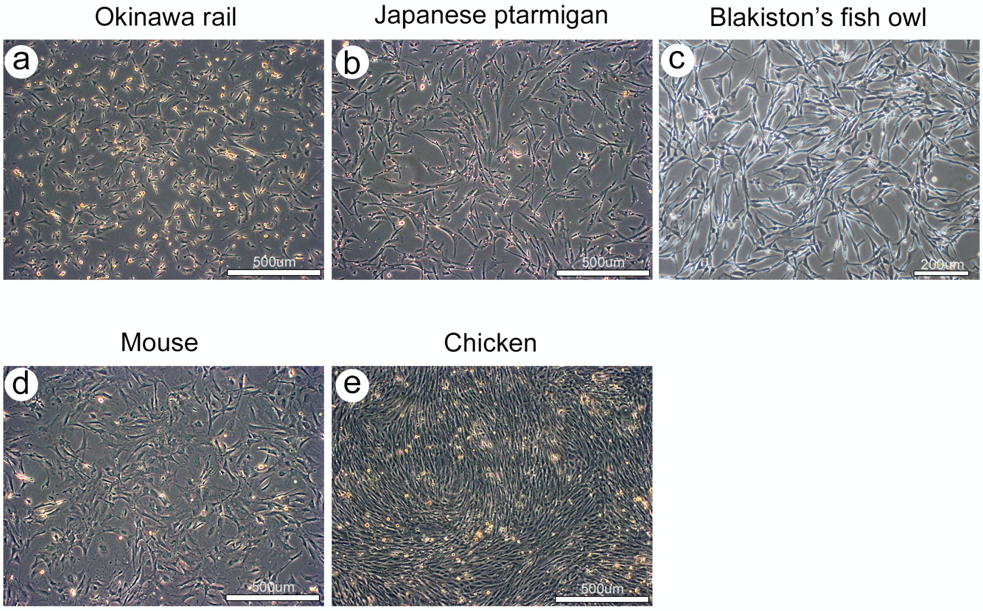

Supplementary Figure 1. Images of primary cells.

Images of the primary cells, including Okinawa rail-derived primary cells (a), Japanese ptarmigan-derived primary cells (b), Blakiston's fish owl-derived primary cells (c), mouse-derived primary cells (d), and chicken-derived primary cells (e). Bars represent 200 µm (c) or 500 µm (a, b, d, e).

a

Species	Reprogramming vector	Establishemnt number	Picked up primary colonies	Establishment clones	Ratio(%)
Mouse	PB-R6F	1st	2	2	100
Chicken	PB-R6F	1st	20	16	80
Okinawa rail	PB-R6F	1st	16	4	25
Japanese ptarmigan	PB-R6F	1st	4	4	100
Blakiston's fish owl	PB-R6F	1st	0	0	0

b

Species	Reprogramming vector	Establishemnt number	Picked up primary colonies	Establishment clones	Ratio(%)
Mouse	PB-TAD-7F	1st	3	3	100
Chicken	PB-TAD-7F	1st	8	8	100
Okinawa rail	PB-TAD-7F	1st	19	8	42.1
		2nd	24	24	100
Japanese ptarmigan	PB-TAD-7F	1st	8	5	62.5
		2nd	32	27	84.4
		3rd	15	11	73.3
Blakiston's fish owl	PB-TAD-7F	1st	7	5	71.4

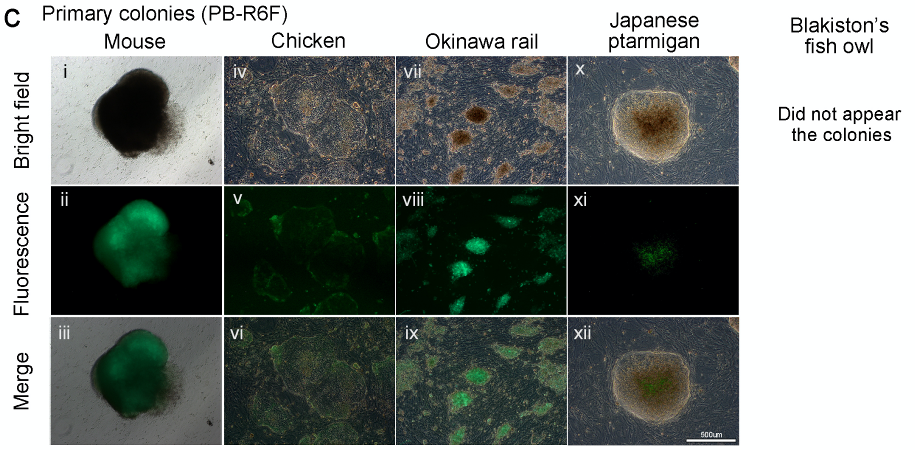

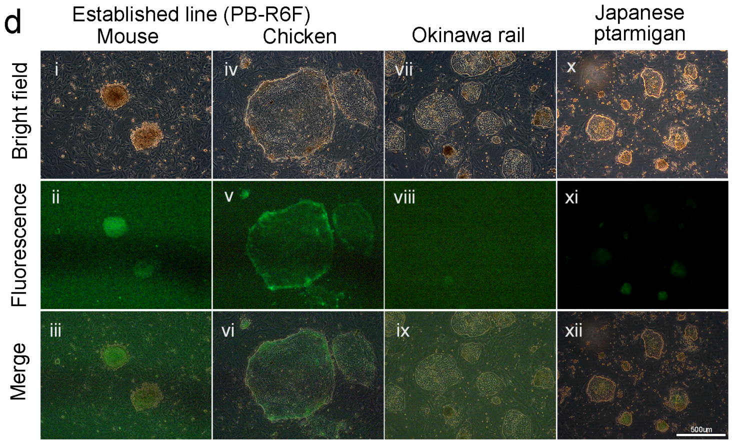

Supplementary Figure 2. Establishment of iPSCs derived from mouse, chicken, Okinawa rail, Japanese ptarmigan, and Blakiston's fish owl using the PB-R6F vector.

a,b: Comparison between PB-R6F and PB-TAD-7F reprogramming vectors for the establishment ratio of avian-derived iPSCs. a: PB-R6F, b: PB-TAD-7F.

c: Primary colonies of iPSCs derived from mice, chickens, Okinawa rail, and Japanese ptarmigans. Panels are bright field images (i, iv, vii, and x), green fluorescence protein (GFP) images (ii, v, viii, xi), and merged images (iii, vi, ix, and xii). Panels show mice (i-iii), panels show chickens (iv-vi), Okinawa rail (vii-ix), and Japanese ptarmigans (x-xii). The bars represent 500 μm.

d: Morphologies of our established iPS cell lines derived from mouse, chicken, Okinawa rail, and Japanese

ptarmigan. Panels are bright field images (i, iv, vii, and x), green fluorescence protein (GFP) images (ii, v, viii, xi), and merged images (iii, vi, ix, and xii). Panels show mice (i-iii), panels show chickens (iv-vi), Okinawa rail (vii-ix), and Japanese ptarmigans (x-xii). The bars represent 500 µm.

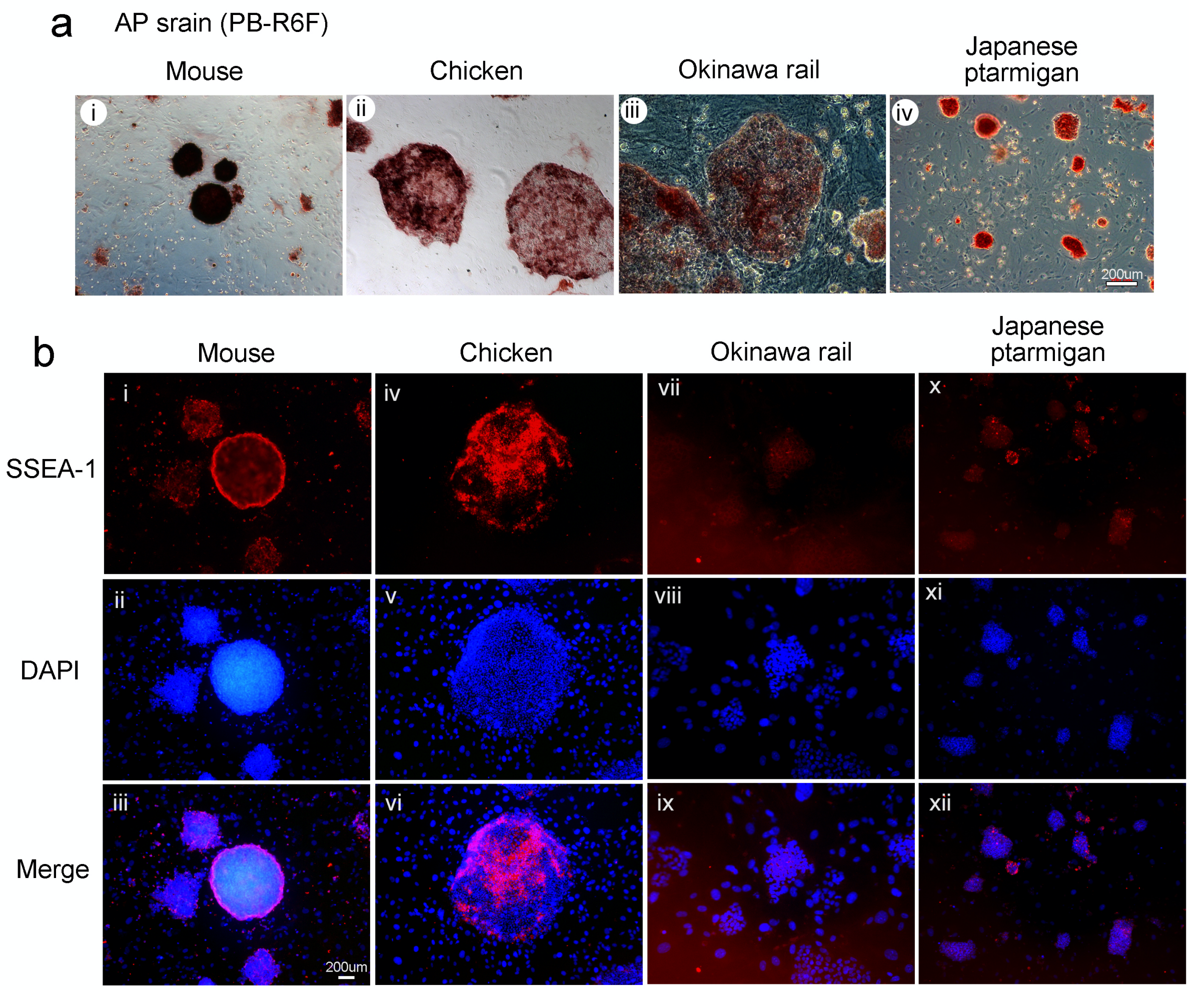

Supplementary Figure 3. Detection of stem cell markers in our established iPSCs derived from mouse, chicken, Okinawa rail, and Japanese ptarmigan using the PB-R6F reprogramming vector.

a: Alkaline phosphatase (AP) activity in iPSCs derived from mouse (i), chicken (ii), Okinawa rail (iii), and Japanese ptarmigan (iv). The bars represent 200 µm.

b: Detection of stage-specific embryonic antigen (SSEA)-1 in mouse (i-iii), chicken (iv-vi), Okinawa rail (vii-ix), and Japanese ptarmigan (x-xii). Panels show SSEA-1 images (i, iv, vii, and x), 4',6-diamidino-2-phenylindole (DAPI) images (ii, v, viii, and xi), and merged images (iii, vi, ix, and xii). The bars represent 200 µm.

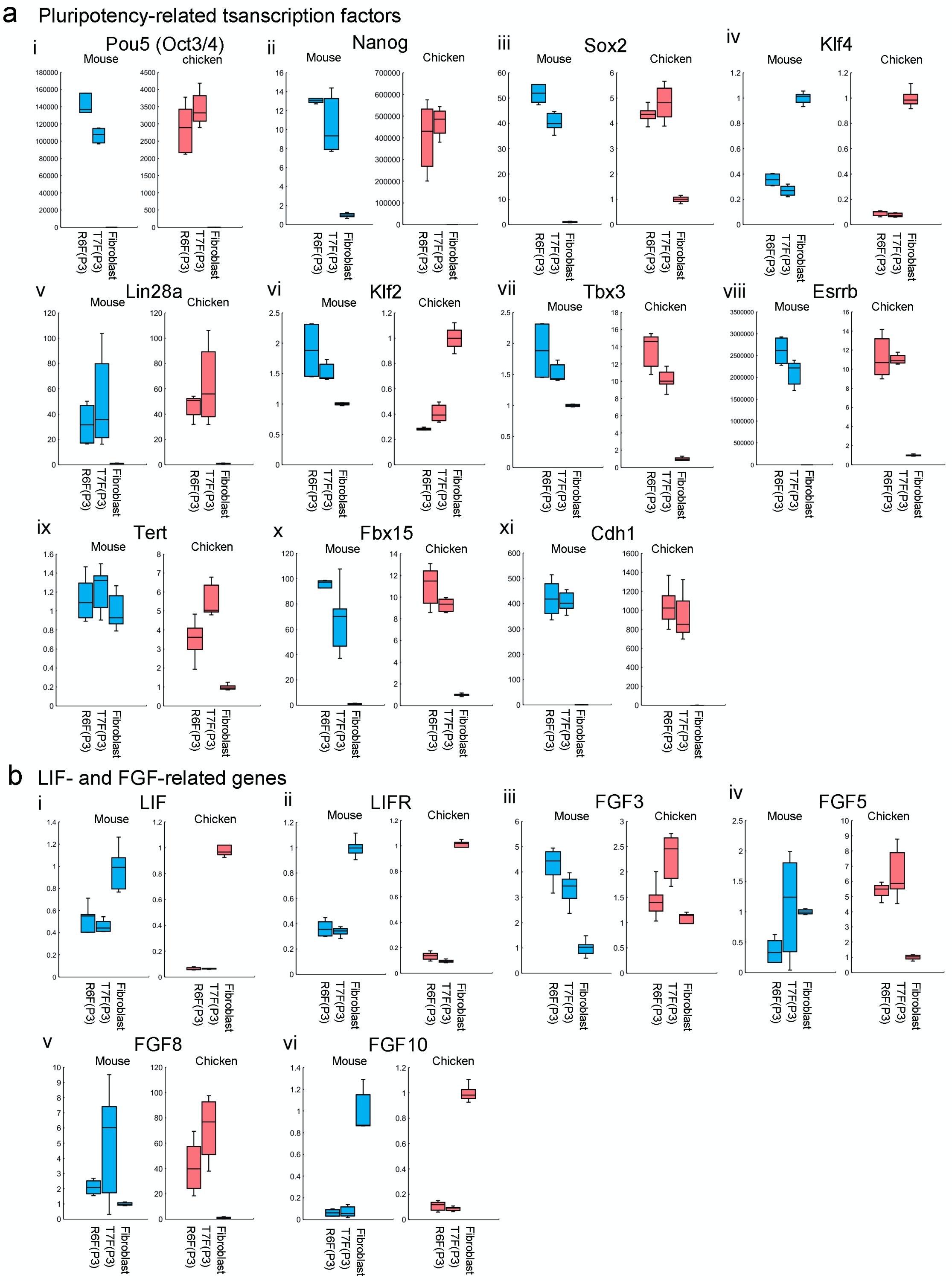

Supplementary Figure 4. Comparison of pluripotency-related gene expression in mouse and chicken iPSCs with PB-R6F, iPSCs with PB-TAD-7F, and fibroblasts.

a: Endogenous expression of pluripotency marker genes in mouse and chicken-derived cells. The mRNA expression of *Pou5* (*Oct3/4*) (i), *Nanog* (ii), *Sox2* (iii), *Klf4* (iv), *Lin28a* (v), *Klf2* (vi), *Tbx3* (vii), *Esrrb* (viii), *Tert* (ix), *Fbx15* (x), *Cdh1* (xi) shows.

b: Endogenous expression of *LIF*- and *FGF*-related genes in mouse and chicken-derived cells. The mRNA

expression of *LIF* (i), *LIFR* (ii), *FGF3* (iii), *FGF5* (iv), *FGF8* (v), and *FGF10* (vi) shows.

Gene expression was quantified relative to *GAPDH* (internal control), and showed that the fibroblast expression level was 1.0. Blue bars denote mouse-derived cells and red bars denote chicken-derived cells. Bars represent iPSCs with PB-R6F (passage number 3; P3), iPSCs with PB-TAD-7F (passage number 3; P3), and fibroblasts. Centerlines of box plots indicate medians; box limits indicate the 25th and 75th percentiles. n=4 (mouse PB-R6F and chicken fibroblast), n=6(other).

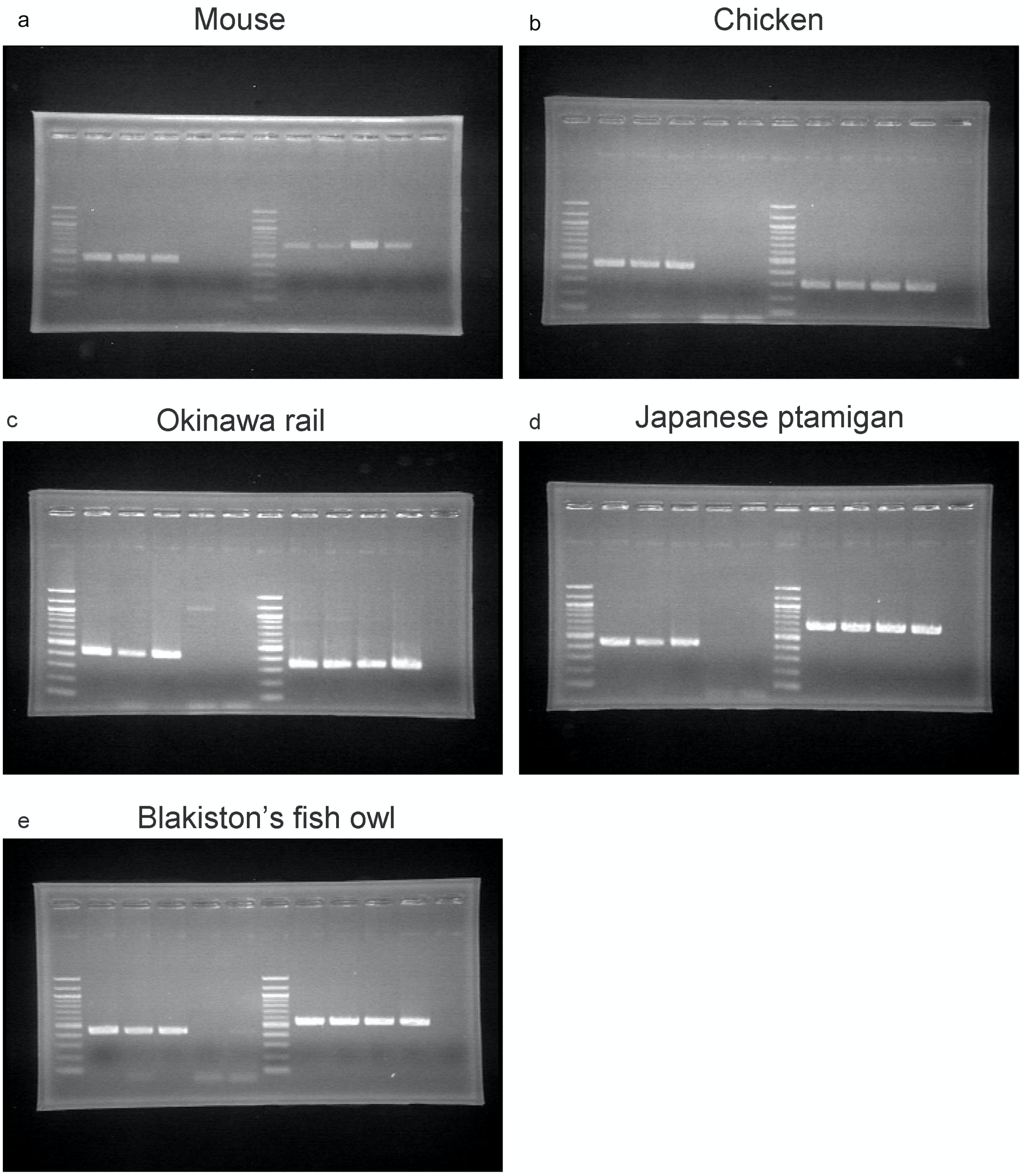

Supplementary Figure 5. Detection of transduced genes by genomic PCR (full scan blot).

The gel photographs show mouse (a), chicken (b), Okinawa rail (c), Japanese ptarmigan (d), and Blakiston's fish owl (e). The six lanes on the left of each gel show the PB-TAD-7F reprogramming vector. The six

right lanes of each gel show *Tsc-2* (internal control). Detailed information is shown in Figure 2a (high-magnification image of these gel).

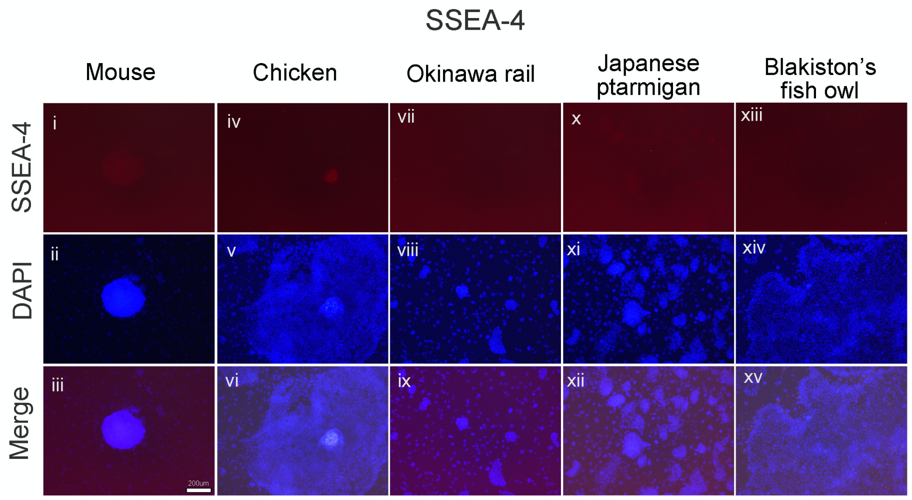

Supplementary Figure 6. Detection of stage-specific embryonic antigen (SSEA)-4.
Images are from mouse (i-iii), chicken (iv-vi), Okinawa rail (vii-iv), Japanese ptarmigan (x-xii), and Blakiston's fish owl (xiii-xv). Panels show SSEA-4 (i, iv, vii, x, xiii), DAPI (ii, v, viii, xi, xiv), and the merged (iii, vi, ix, xii, xv) images. The bars represent 200 µm.

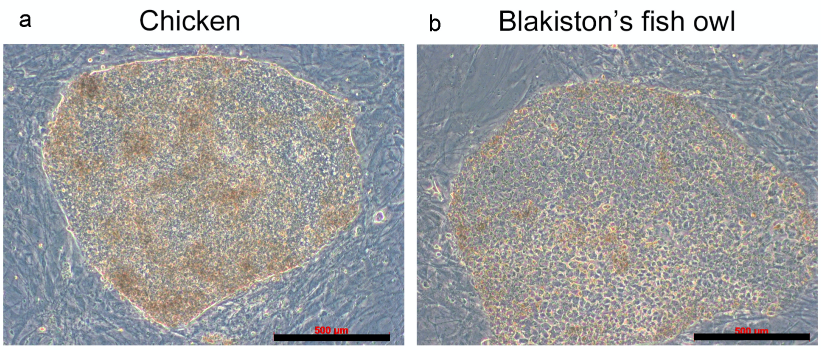

Supplementary Figure 7. Morphology of iPSCs from chick and Blakiston's fish owl at a late passage.
The images are a chicken iPSC colony at passage 20 (a) and a Blakiston's fish owl iPSC colony at passage 20 (b). The bars represent 500 µm.

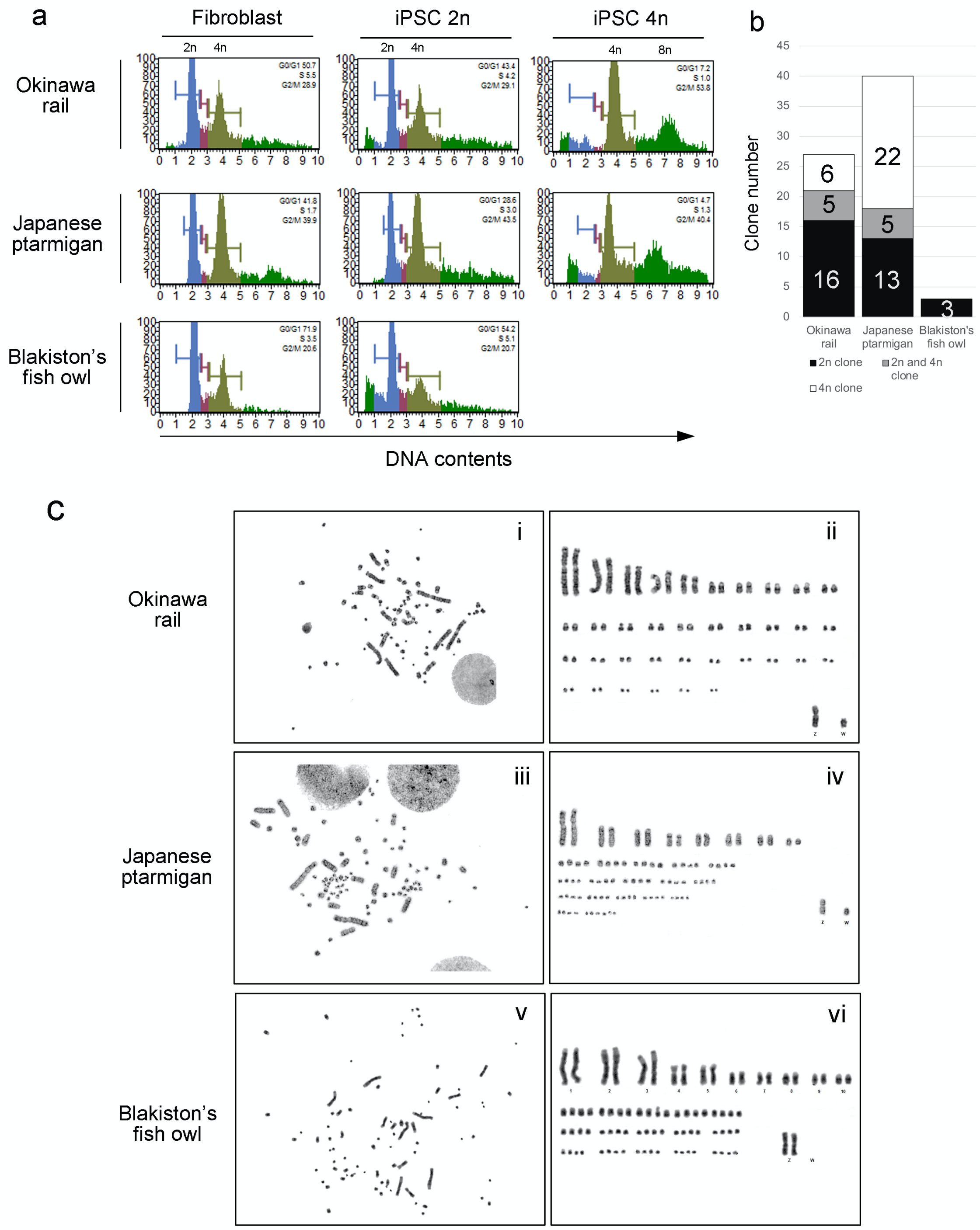

Supplementary Figure 8 Chromosomal analysis of Okinawa rail, Japanese ptarmigan, and Blakiston's fish owl-derived iPSCs.

a: DNA content profile analysis of Okinawa rail, Japanese ptarmigan, and Blakiston's fish owl-derived iPSCs by flow cytometry. The DNA content profile was analyzed by flow cytometry to evaluate chromosomal polyploidy and diploidy. Panels represent fibroblasts, diploid iPSCs, and polyploid iPSCs.

b: Number of diploid and polyploid clones derived from Okinawa rail, Japanese ptarmigan, and Blakiston's fish owl-derived iPSCs. The black area shows the number of 2n clones, the gray area shows the number of 2n and 4n clones, and the white area shows the number of 4n clones.

c: Chromosomal patterns of endangered Japanese avian-derived iPSCs. The panels show the chromosome pattern of Okinawa rail-derived iPSCs (i, ii), Japanese ptarmigan-derived iPSCs (iii, iv), and Blakiston fish owl-derived iPSCs (v, vi). Panels show the representative mitotic phase (i, iii, v), and the aligned chromosomes (ii, iv, vi).

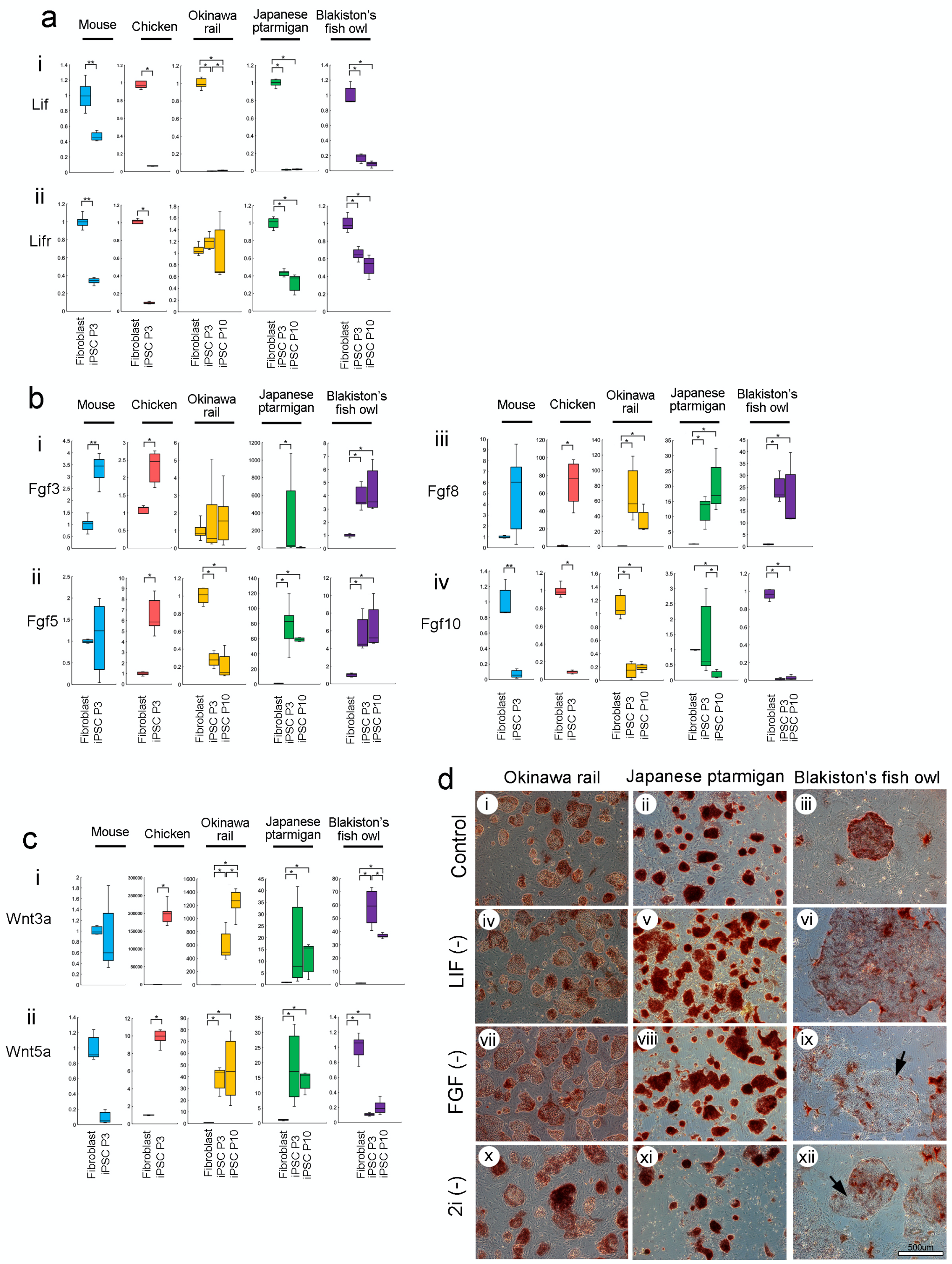

Supplementary Figure 9 *Lif*, *Lifr*, *Fgf* family, and *Wnt* gene expression and Cell culture conditions of Okinawa rail, Japanese ptarmigan, and Blakiston's fish owl-derived iPSCs.

a: *Lif* (i) and *Lifr* (ii) gene expression in mouse, chicken, Okinawa rail, Japanese ptarmigan, and Blakiston fish owl-derived iPSCs.

b: *Fgf 3* (i),*Fgf 5* (ii), *Fgf 8* (iii), *Fgf 10* (iv) gene expression in mouse, chicken, Okinawa rail, Japanese ptarmigan, and Blakiston fish owl-derived iPSCs.

c: *Wnt 3a* and *Wnt 5a* gene expression in mouse, chicken, Okinawa rail, Japanese ptarmigan, and Blakiston fish owl-derived iPSCs.

Gene expression was quantified relative to that of the *GAPDH* internal control. n=4 (chicken firboblast), n=6 (other). * P<0.05, ** P<0.01. Blue bars, mouse; red bars, chicken; yellow bars, Okinawa rail; green bars, ptarmigan; and purple bars, Blakiston's fish owl-derived cells. Mouse and chicken: bars represent fibroblasts and iPSCs (passage number 3; P3). Okinawa rail, Japanese ptarmigan, and Blakiston's fish owl: bars represent fibroblasts, iPSCs (P3), and iPSCs (P10). Centerlines of box plots indicate medians; box limits indicate the 25th and 75th percentiles.

d: Alkaline phosphatase staining of Okinawa rail (i, iv, vii, x), Japanese ptarmigan (ii, v, viii, xi), and Blakiston's fish owl iPSCs (iii, vi, ix, xii) in standard medium (i-iii), LIF minus medium (iv-vi), FGF minus medium (vii-ix), and 2i minus medium (x-xii). The panels are Okinawa rail, Japanese ptarmigans, and Blakiston's fish owls iPSCs. The bars represent 500 μm.

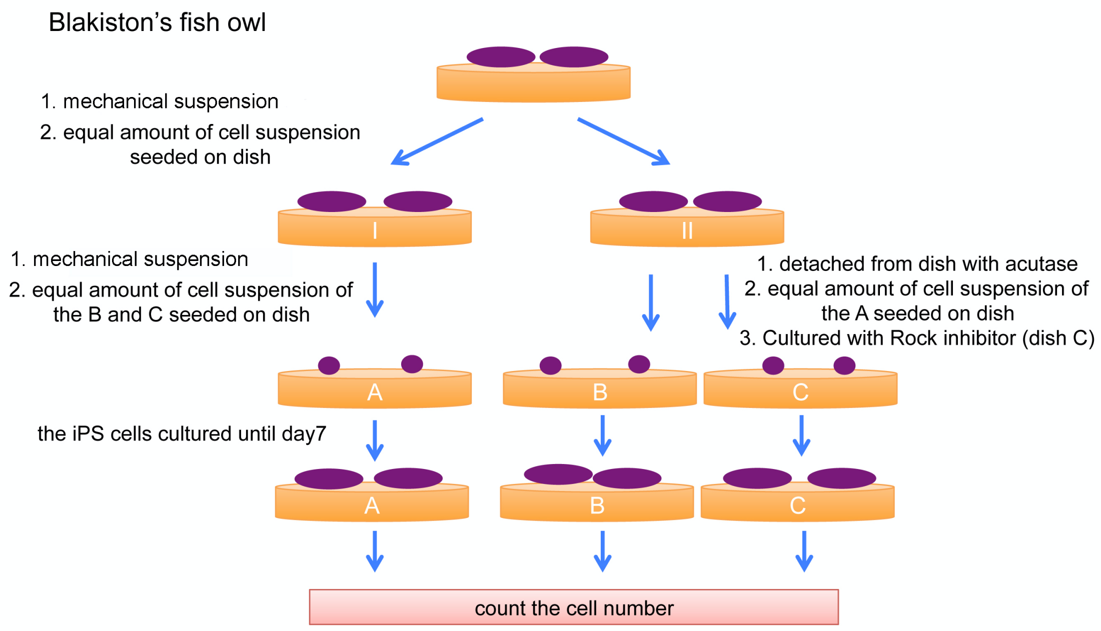

Supplementary Figure 10. **Schematic of cell number counting for Blakiston's fish owl-derived iPSCs.**

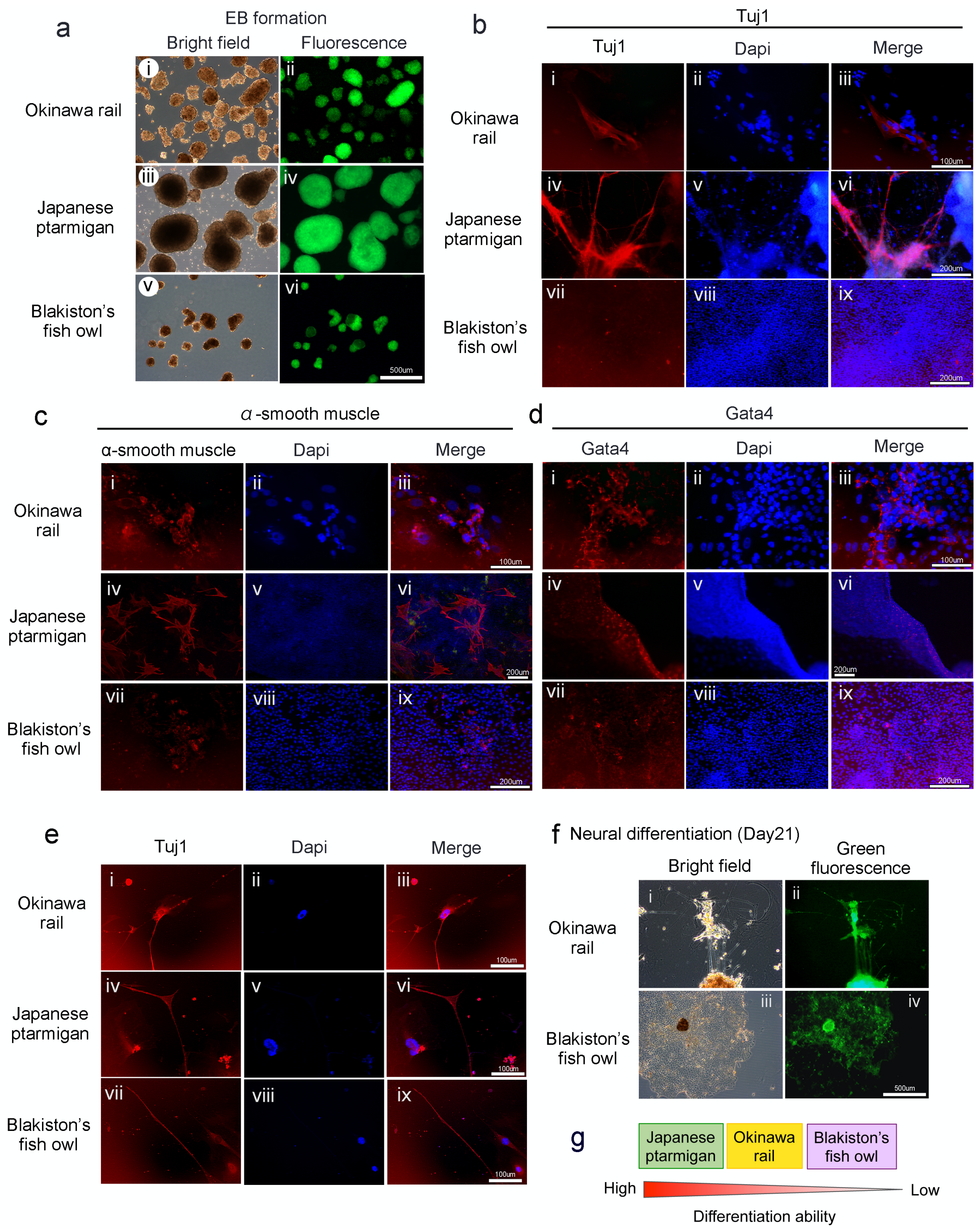

Supplementary Figure 11. ***In vitro*** **differentiation of Okinawa rail, Japanese ptarmigan, and Blakiston's fish owl-derived iPSCs.**

a: Cytomorphology of embryoid bodies (EBs). Panels show Okinawa rail-derived EBs (i, ii), Japanese ptarmigan-derived EBs (iii, iv), and Blakiston's fish owl-derived EBs (v, vi). The panels represent bright field images (i, iii, v) and GFP (ii, iv, vi). The bars represent 500 μm.

b, c, and d: Immunocytochemical evaluation of the ability to differentiate into the three germ layers in vitro. Ectodermal cells (Tuj1) (b), mesodermal cells (alpha-smooth muscle) (c), and endodermal cells

(Gata4) (d) are shown. Panels show Okinawa rail-derived cells (i-iii), Japanese ptarmigan-derived cells (iv-vi), and Blakiston's fish owl-derived cells (vii-ix). The bars represent 100 μm (Okinawa rail) or 200 μm (Japanese ptarmigan and Blakiston fish owl). Red fluorescence (i, iv,vii), dapi (ii, v, viii) and merge (iii, vi, ix).

e: Cytomorphology of dendrite-like structures derived from Okinawa rail, Japanese ptarmigan, and Blakiston's fish owl-derived iPSCs. Panels show Okinawa rail-derived cells (i-iii), Japanese ptarmigan-derived cells (iv-vi), and Blakiston's fish owl-derived cells (vii-ix). The bars represent 100 μm (Okinawa rail) or 200 μm (Japanese ptarmigan and Blakiston fish owl). Red fluorescence (i, iv,vii), dapi (ii, v, viii) and merge (iii, vi, ix). Bars represent 100 μm.

f: Differences in the *in vitro* differentiation potential of Okinawa rail and Blakiston fish owl-derived iPSCs at day 21. Panels show Okinawa rail (i, ii) and Blakiston's fish owls (iii, iv). Panels show bright field (i, iii) and green fluorescence (ii, iv). The bars represent 500 μm.

g: Differentiation ability of Okinawa rail, Japanese ptarmigan, and Blakiston fish owl-derived iPSCs. Differences in *in vitro* differentiation potential among Okinawa rail, Japanese ptarmigan, and Blakiston fish owl-derived iPSCs. Japanese ptarmigan-derived iPSCs have the highest differentiation ability, followed by Okinawa rail-derived iPSCs and Blakiston's fish owl-derived iPSCs.

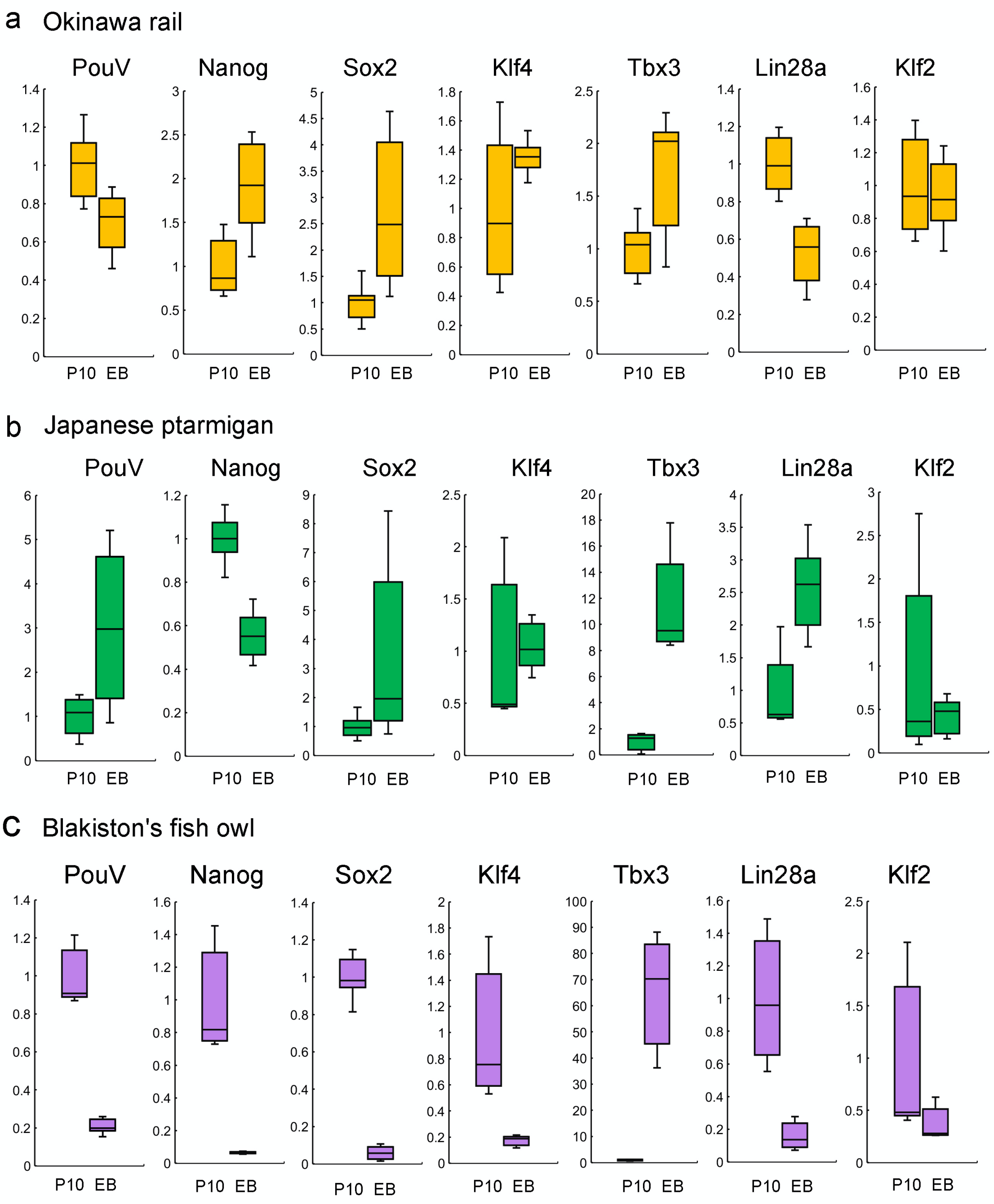

Supplementary Figure 12. Comparison of pluripotency-related gene expression of Okinawa rail, Japanese ptarmigan, and Blakiston's fish owl-derived cells between iPSCs with PB-TAD-7F and embryoid bodies (EBs).

a: Endogenous expression of pluripotency marker genes in Okinawa rail-derived iPSCs and EBs.

b: Endogenous expression of pluripotency marker genes in Japanese ptarmigan-derived iPSCs and EBs.

c: Endogenous expression of pluripotency marker genes in Japanese ptarmigan-derived iPSCs and EBs.

Gene expression was quantified relative to *GAPDH* (internal control). The expression level is 1.0 in iPSCs with PB-TAD-7F (passage 10; P10). The bars represents iPSCs with PB-TAD-7F (P10) and EBs. n=6. Centerlines of box plots indicate medians; box limits indicate the 25th and 75th percentiles.

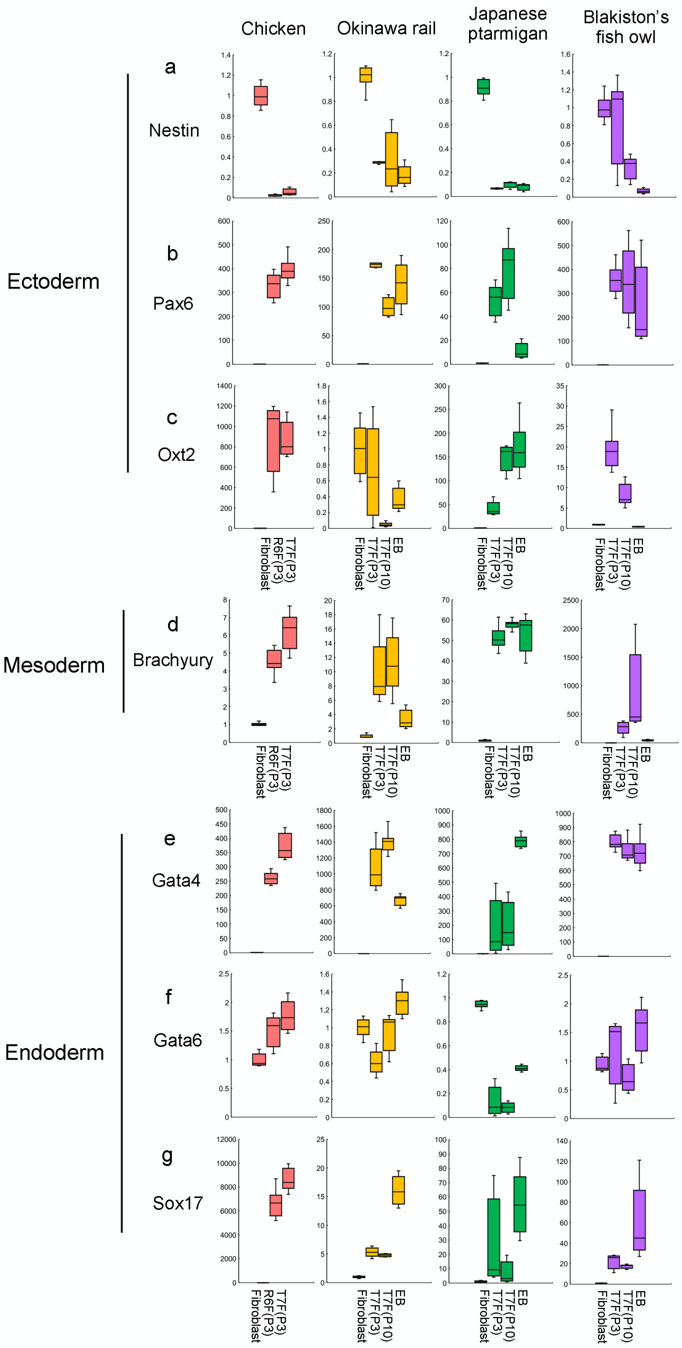

Supplementary Figure 13. Comparison of the germ layer-related gene expression of chick, Okinawa rail, Japanese ptarmigan, and Blakiston's fish owl-derived cells among fibroblasts, iPSCs (passage 3; P3), iPSCs (passage 10, P10), and embryoid bodies (EBs).

The mRNA expression of Nestin (a), Pax6 (b), Oxt2 (c), Brachyury (d), Gata4 (e), Gata6 (f), Sox17 (g) shows. Chicken (red), Okinawa rail (yellow), Japanese ptarmigan (green), Blakiston's fish owl (purple). The bars represent fibroblasts, iPSCs (P3), iPSCs (P10), and EBs. n=6. Centerlines of box plots indicate medians; box limits indicate the 25th and 75th percentiles.

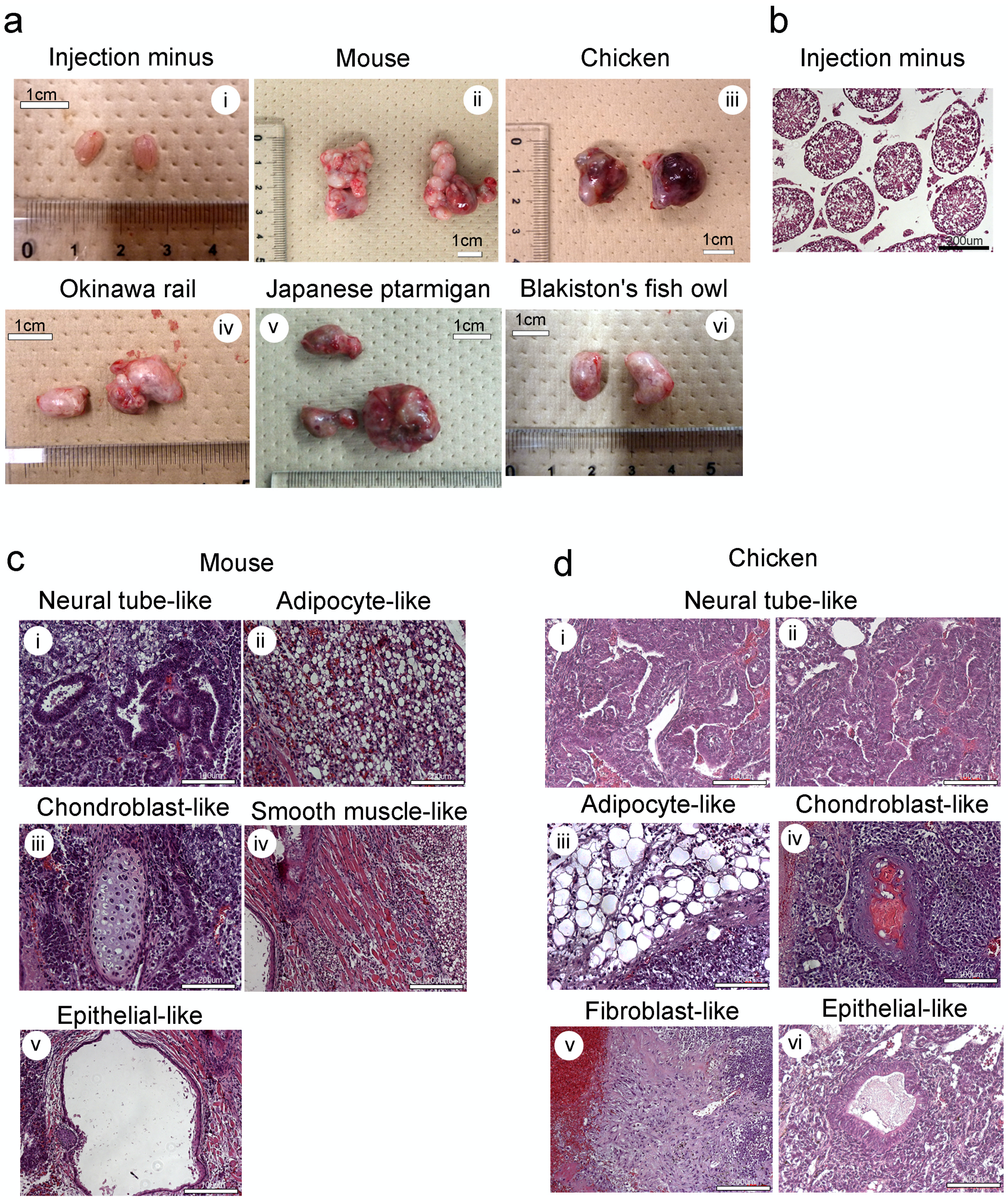

Supplementary Figure 14. Differentiation of the established mouse, chicken, Okinawa rail, Japanese ptarmigan, and Blakiston's fish owl-derived iPSCs *in vivo*.

a: Teratoma formation from iPSCs generated with the PB-TAD-7F reprogramming vector.

Injection minus (control) (i), mouse iPSC-derived teratomas (ii); chicken iPSC-derived teratomas (iii); Okinawa rail iPSC-derived teratomas (iv); Japanese ptarmigan iPSC-derived teratomas (v); Blakiston's fish owl iPSC-derived teratomas (vi). Bars show 1cm.

b–d: Histological analysis of injection minus (b), mouse iPSC-derived teratomas (c), and chicken iPSC-derived teratomas (d). Various tissues originating from the three germ layers were identified, and include neural tube-like structures (ectoderm) (ci, di, dii), adipocyte-like (cii, diii), fibroblast-like (dv), chondroblast-

like (ciii, div), and smooth muscle-like structures (civ) (mesoderm), and an epithelial-like structure (cv, dvi) (endoderm). The tissues were stained with hematoxylin and eosin. Bars represent 100–200 µm.

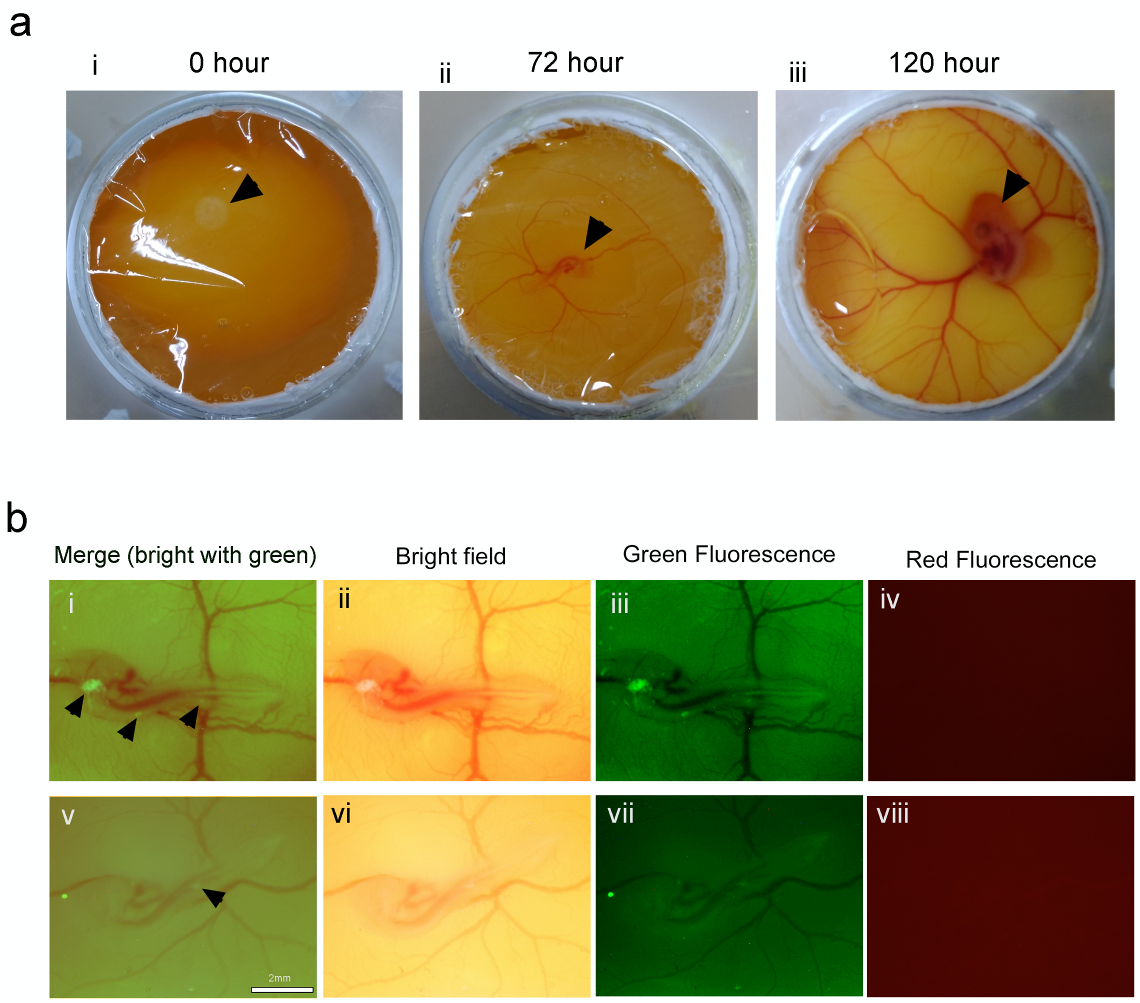

Supplementary Figure 15. Formation of the chimeric embryo after injection of Japanese ptarmigan-derived iPSCs into the chicken blastoderm.

a: The developmental process of Japanese ptarmigan-derived iPSCs injected into chicken embryos. The panels show the time after injection: 0 h (i), 72 h (ii), and 120 h (iii).

b: Embryos exhibited iPSC contribution after injection. The arrow indicates green signal. Panels represent bright field and green fluorescence image (i, v), bright field (ii, vi), green fluorescence (iii, vii), and red fluorescence (iv, viii). Bars represent 2 mm.

a PB-TAD-7F

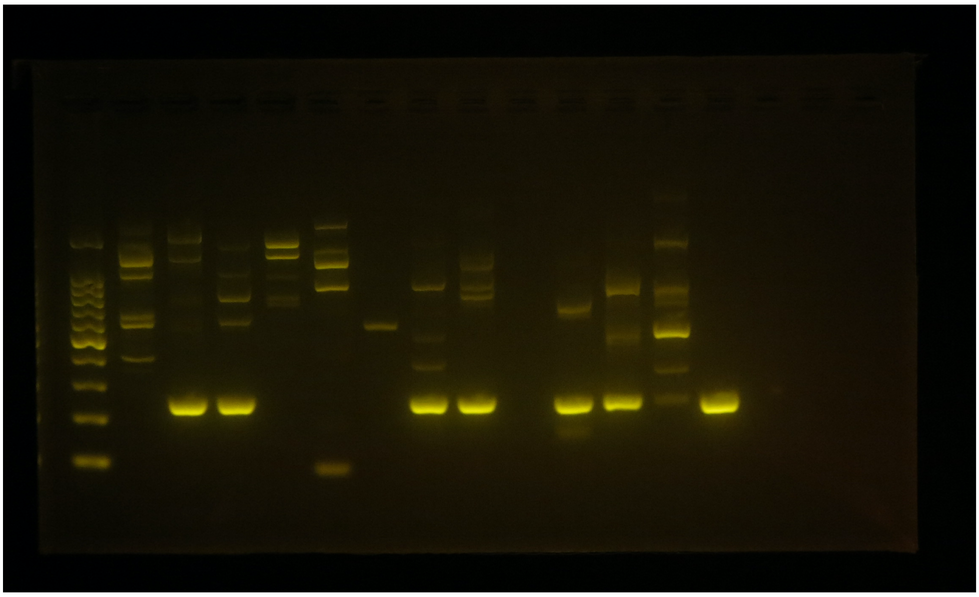

b Tsc-2 (internal control)

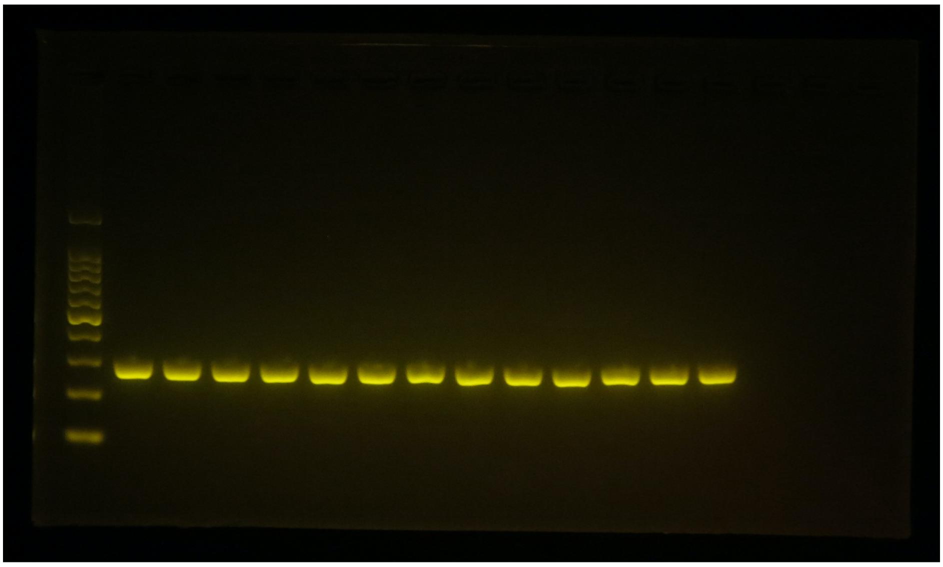

Supplementary Figure 16 Detection of transduced genes by genomic PCR (full scan blot).

a: Reprogramming vector for PB-TAD-7F; b: Tsc-2 gene (internal control). Detailed information can be found in Figure 8f (a high-magnification image of this gel).

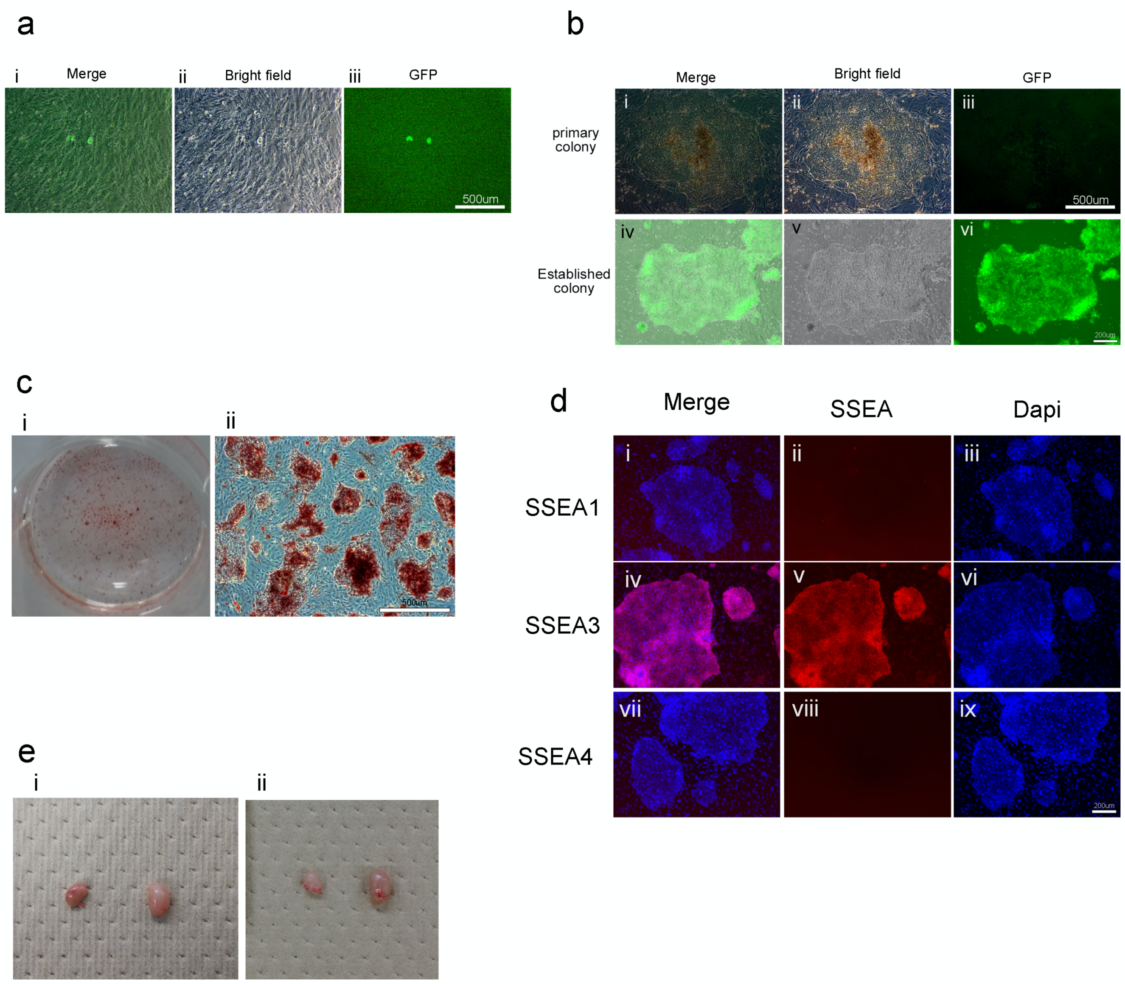

Supplementary Figure 17 Establishment of Japanese golden eagle reprogrammed cells with PB-TAD-7F reprogramming vector.

a: Transduction of the reprogramming vector into Japanese golden eagle cells after 72 h. The panels show a merged image (i), a bright field image (ii), and a GFP image (iii). The bar indicates 500 µm.

b: Morphological features of Japanese golden eagle-derived primary and established iPSC colonies using PB-TAD-7F. The panels show a primary colony of reprogrammed cells using PB-TAD-7F (i-iii) and iPSC colonies established using PB-TAD-7F (iv-vi). Panels show merge images (i, iv), bright field images (ii, v), and GFP images (iii, vi). The bars indicate 500 µm (primary colony) and 200 µm (established colony).

c: Alkaline phosphatase staining of Japanese golden eagle iPSCs established using PB-TAD-7F (i, ii). The bar indicates 500 µm.

d: Pluripotency marker staining. The panels show SSEA-1 (i-iii), SSEA-3 (iv-vi), and SSEA-4 (vii-ix). The panels show merged images (i, iv, vii), red fluorescence (iv, v, vi), and counterstained with DAPI (iii, vi, ix). Bar indicates 200 µm.

e: Macroscopic analysis of tumors formed in the testis tissue of SCID mice derived from Japanese golden eagle iPSCs. The images show the testicular tissues of SCID mice with iPSCs established from PB-TAD-7F (i, ii).

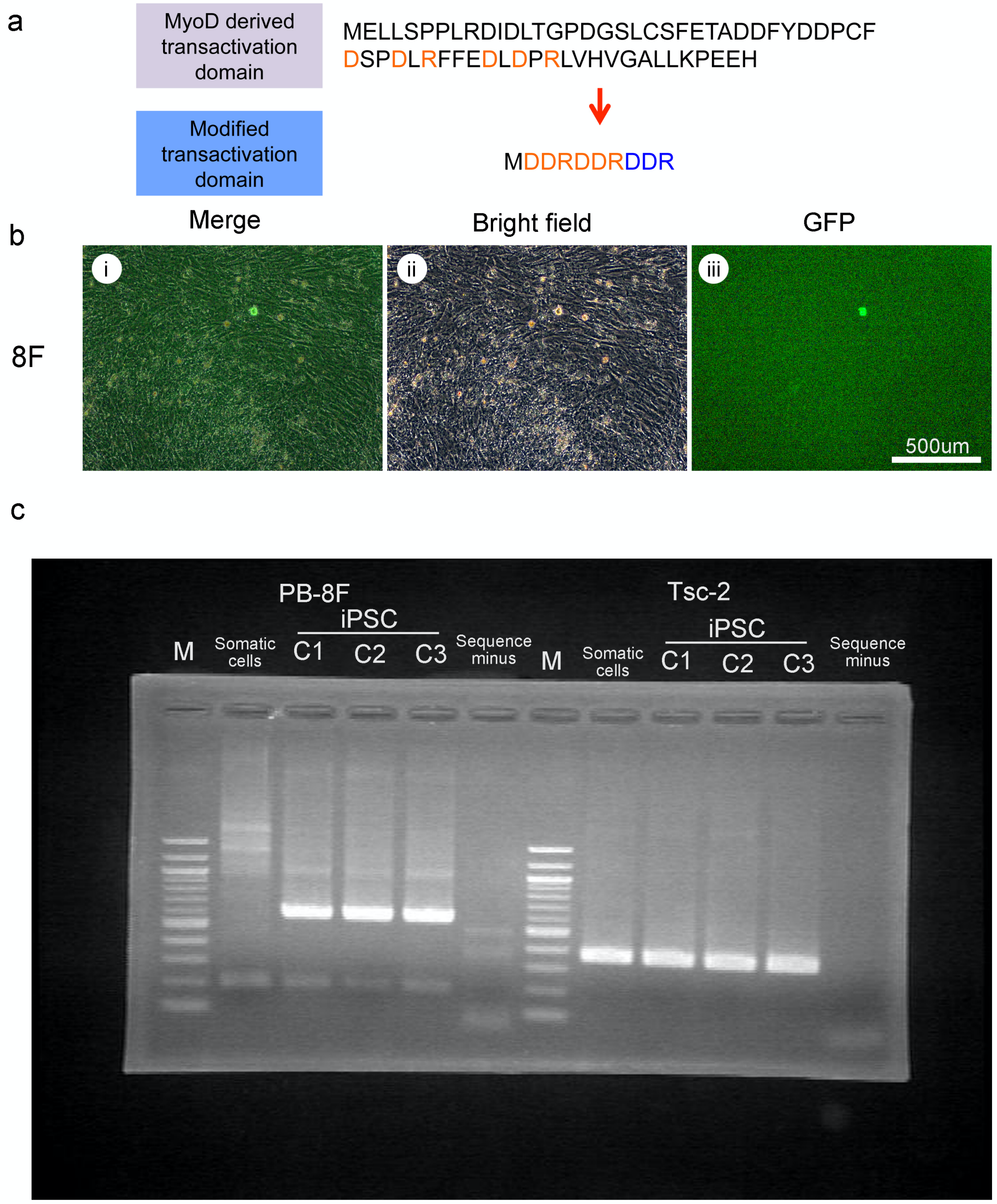

Supplementary Figure 18. Establishment of Japanese golden eagle iPSCs using PB-DDR-8F reprogramming vector.

a: Amino acid sequence of the MyoD-derived transactivation domain (the domain used in the PB-TAD-7F vector) and modified transactivation domain (the domain used in the PB-DDR-8F vector).

b: Transduction of the reprogramming vector into Japanese golden eagle cells after 72 h. Panels show transduction of the PB-DDR-8F reprogramming vector. The panel shows the merged image (i), a bright fields image (ii), and GFP image (iii). The bar indicates 500 μm.

c: Detection of the reprogramming cassettes of PB-DDR-8F by genomic PCR. The left six lanes show

detection of the PB-DDR-8F vector. The six right lanes show the detection of the *Tsc-2* gene (internal control).

DDR-DDR-DDR-8F

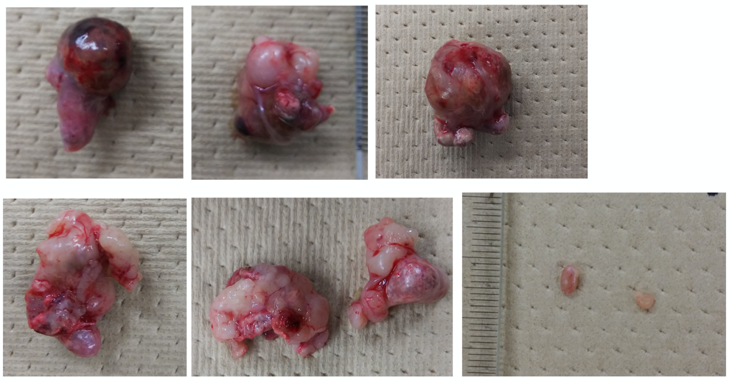

Supplementary Figure 19. Macroscopic analysis of tumors formed in testis tissue of SCID mice from Japanese golden eagle iPSCs.

Images show testis tissues of SCID mice with iPSCs established from PB-DDR-8F.

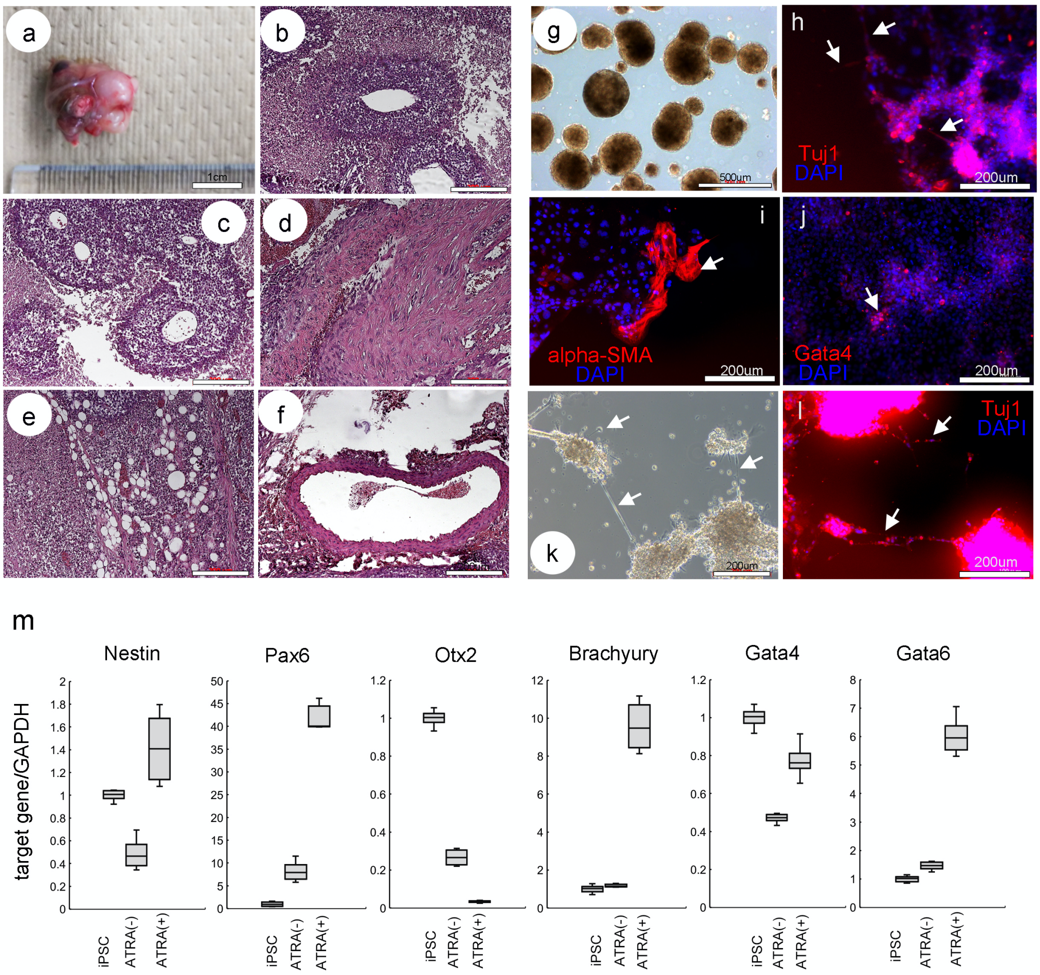

Supplementary Figure 20. Differentiation ability of established Japanese golden eagle-derived iPSCs.

a: Representative appearance of tumors in the testes of SCID mice. The bar indicates 1 cm.

b–f: Histological analysis of the tumors formed in the testis tissue of SCID mice. Shown are neural tube-like structures (b and c; ectoderm), fibroblast-like tissue (d, mesoderm), adipocyte-like tissue (e, mesoderm), and epithelial-cell-like tissue (f, endoderm). Bars indicate 200 µm.

g–l: Immunocytochemical evaluation of the in vitro ability to differentiate into the three germ layers. Shown are ectodermal markers (Tuj1 and g), mesoderm markers (alpha-SMA, h), and endoderm marker (Gata4; i). Bars indicate 200 µm. (j) Cytomorphology of the Japanese golden eagle iPSC-derived EBs. Bars indicate 500 µm. (k l) In vitro differentiation of Japanese golden eagle iPSCs in ATRA+ medium. (k) Cytomorphology of neuron-like cells. (l) Immunological staining for the neuron-like cell marker (Tuj1). Bars indicate 200 µm.

m: Relative mRNA expression of three germ layer differentiation-related genes evaluated by quantitative RT-PCR in differentiated cells. Bars show gene expression in the Japanese golden eagle iPSCs, differentiated cells in ATRA medium, and differentiated cells in ATRA+ medium. *: $P<0.05$. Gene expression was quantified relative to that of the *GAPDH* internal control. n=6.

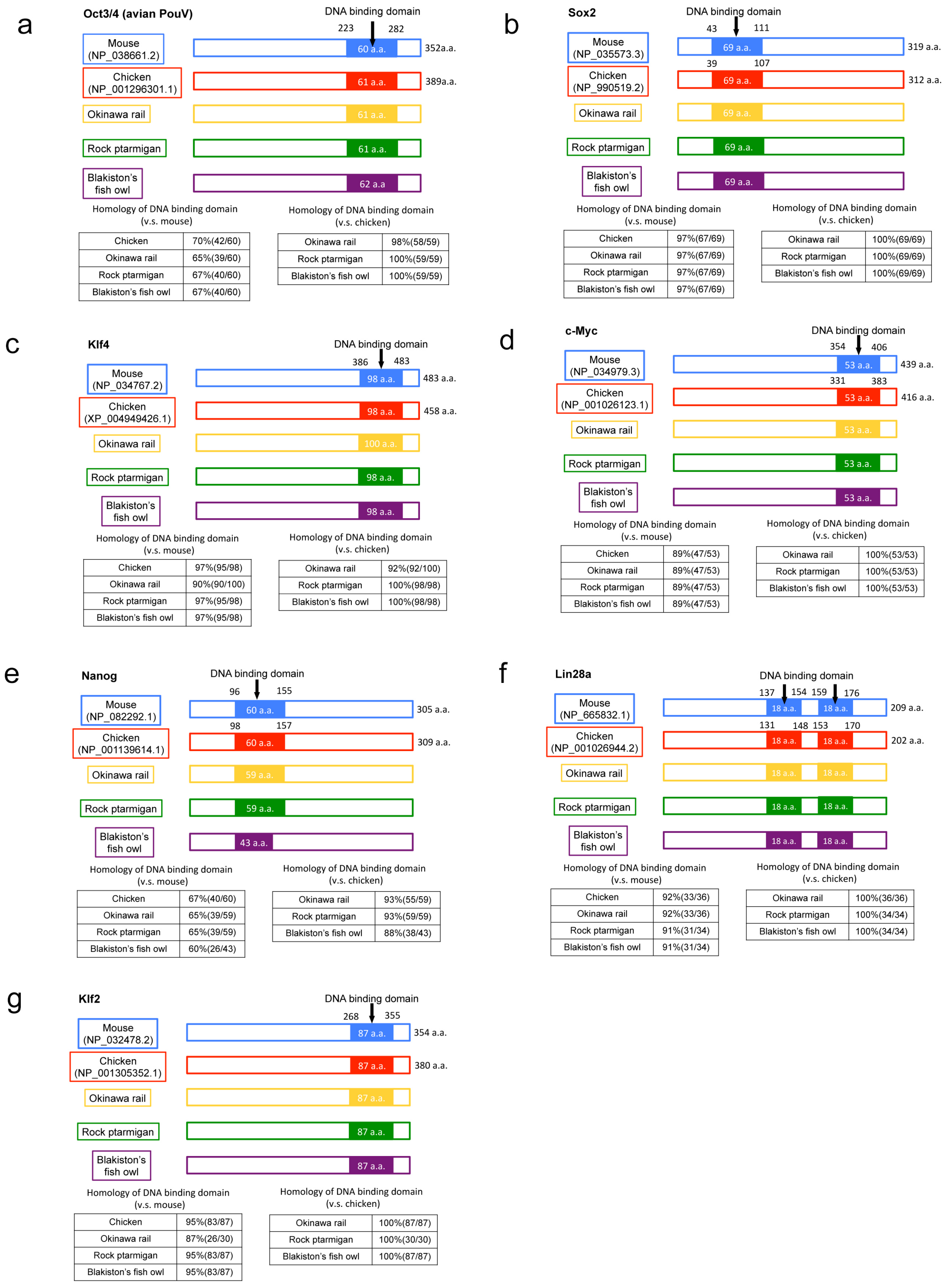

Supplementary Figure 21. Comparison of amino acid homologies of the DNA binding domains of the seven reprogramming factors.

a: Oct3/4 (avian POU5), b: Sox2, c: Klf4, d: c-Myc, e: Nanog, f: Lin28a, g: Klf2

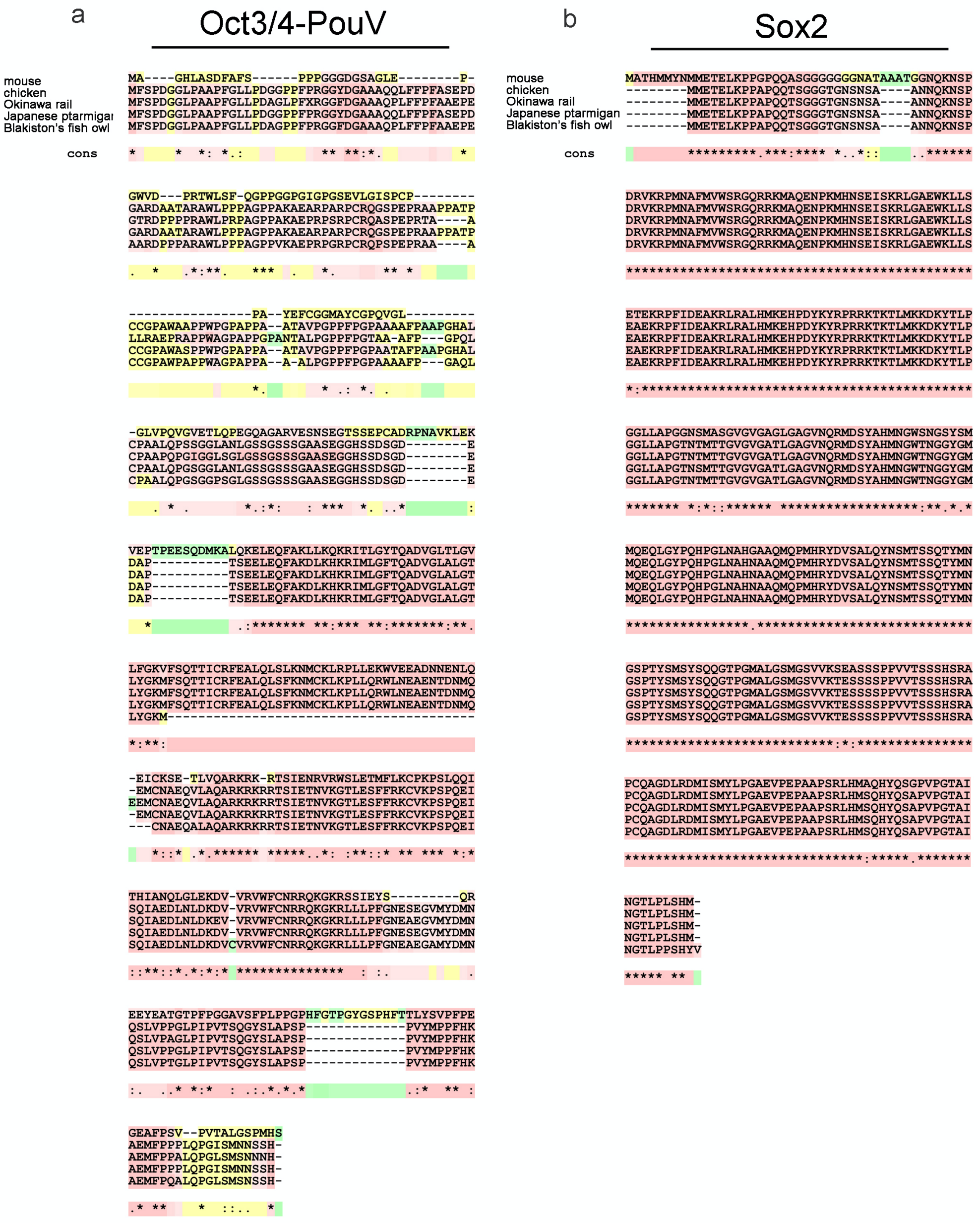

Supplementary Figure 22. Comparison of amino acid homologies for Oct3/4 (Avian: Pou5) and Sox2 among mouse, chicken, Okinawa rail, Japanese ptarmigan, and Blakiston's fish owl.

Comparison of amino acid homologies for Oct3/4 (a) and Sox2 (b).

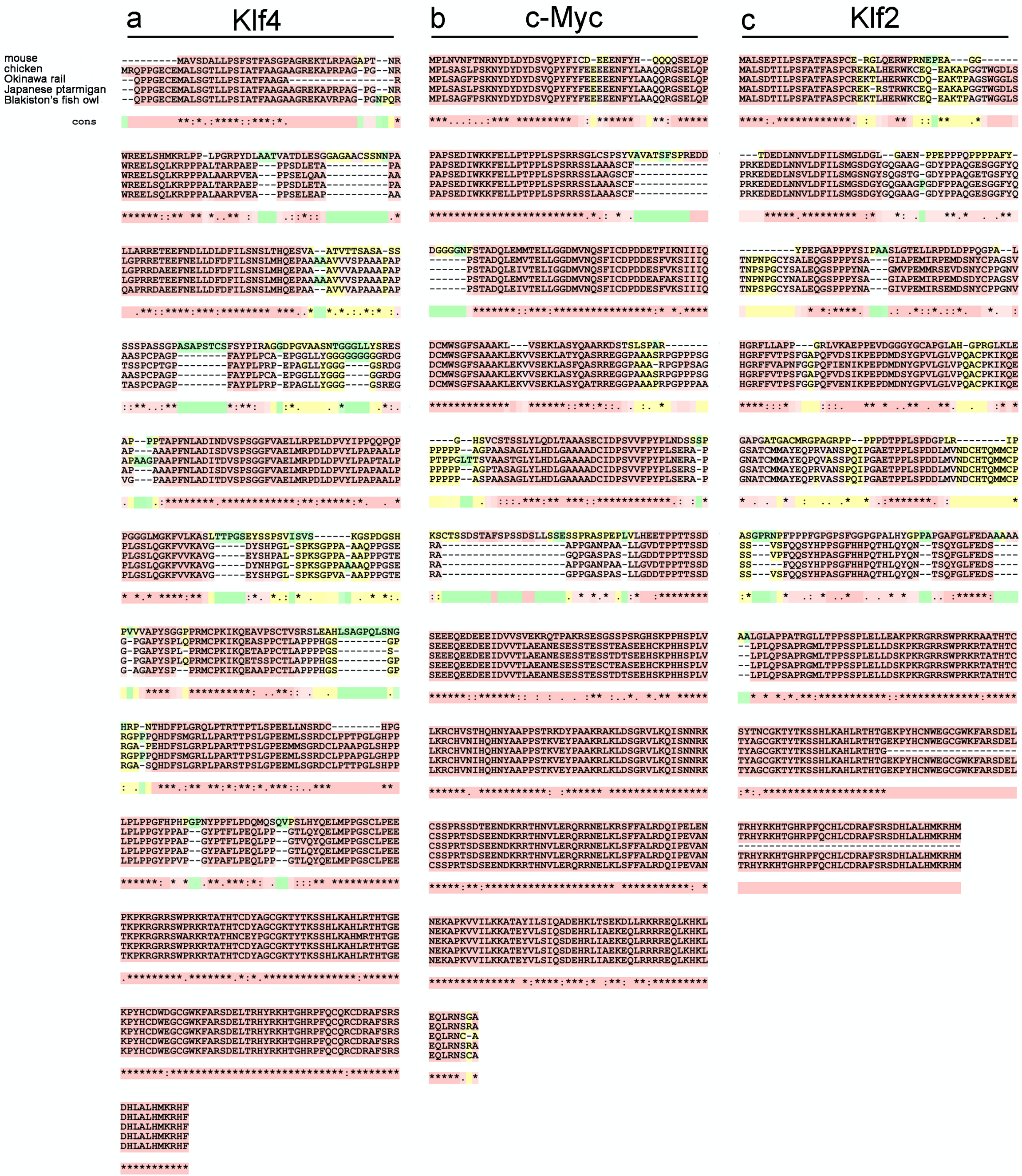

Supplementary Figure 23. Comparison of amino acid homologies for Klf4, c-Myc, and Klf2 among mouse, chicken, Okinawa rail, Japanese ptarmigan, and Blakiston's fish owl.

Comparison of the amino acid homology of Klf4 (a), c-Myc (b), and Klf2 (c).

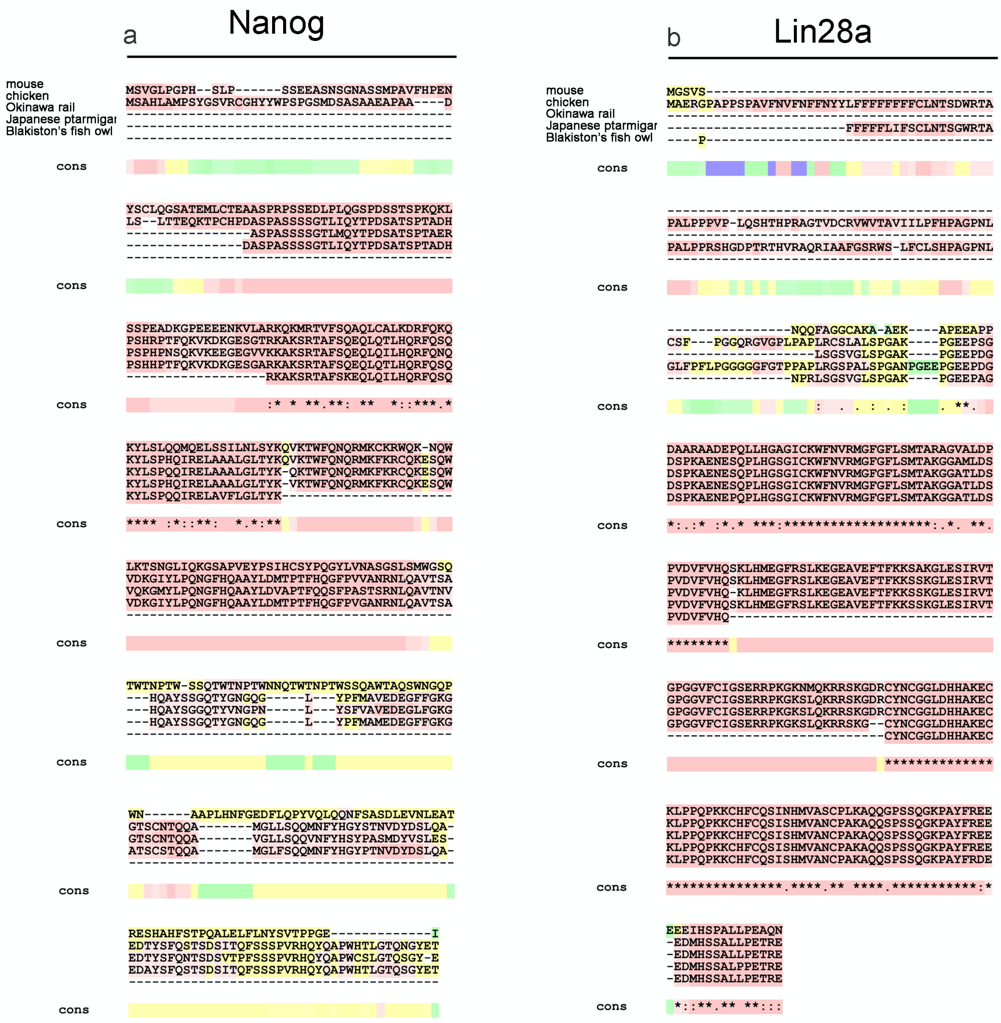

Supplementary Figure 24. Comparison of amino acid homologies for Nanog and Lin28a among mouse, chicken, Okinawa rail, Japanese ptarmigan, and Blakiston's fish owl.

Comparison of amino acid homologies of Nanog (a), and Lin28a (b).

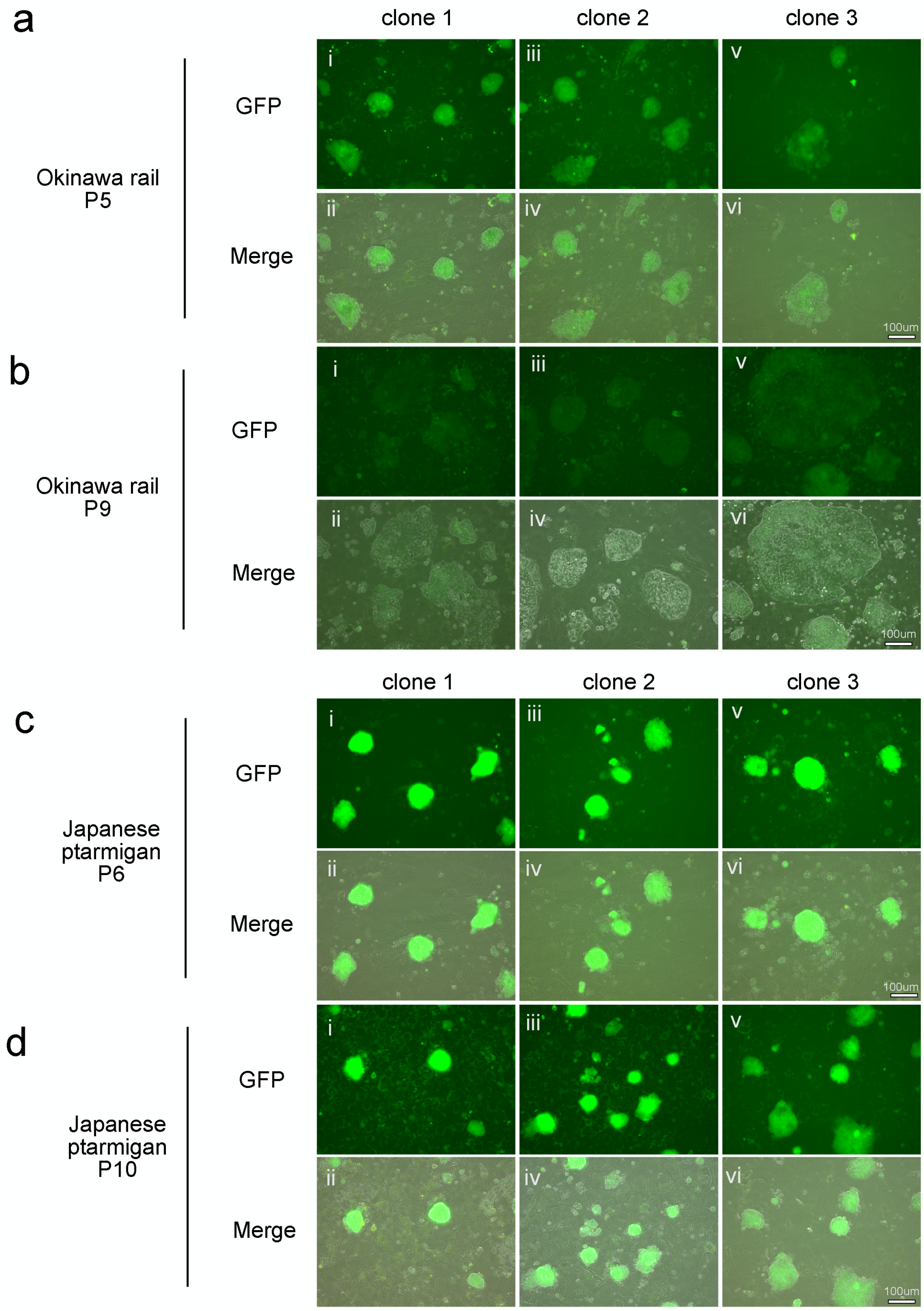

Supplementary Figure 25 Exogeneous gene expression of Okinawa rail and Japanese ptarmigan.

a: Okinawa rail iPSCs passage 5 day7 after the previous reseeded. b: Okinawa rail iPSCs passage 9 day4 after the previous reseeded. c: Japanese ptarmigan iPSCs passage 6 day7 after the previous reseeded. d: Japanese ptarmigan iPSCs passage 10 day4 after the previous reseeded. Panels show clone 1 (i, ii), clone 2 (iii, iv), and clone 3 (v, vi). The images are GFP (i, iii, v), and merge image (ii, iv, vi). Bars show 100 μm.

Supplementary Table 1 Karyotype analysis of Okinawa rail, Japanese ptarmigan, and Blakiston's fish owl.

Species	Clone No.	Chromosome number																					total
		70	71	72	73	74	75	76	77	78	79	80	81	82	83	84	85	86	87	88	89	90	
Okinawa rail	1							1	5	9	13	10	5	3	3	1							50
	2	1	2	2	3	6	6	8	7	8	4	1		2									50
Japanese ptarmigan	1												5	10	6	9	6	5	1	6	2		50
	2											5	5	8	5	3	4	6	4	10			50
Blakiston's fish owl	1							2	9	17	14	5	2	1									50
	2										4	3	2	41									50

Supplementary Table 2 Antibody information

name	Company	Isotype	Cat No.	Dilution
SSEA-1	STEMGENT, Cambridge, MA, US	Mouse IgM	09-0005	1:200
SSEA-3	Bioss, Boston, MA, US	Rabbit IgG	bs-3575R	1:200
SSEA-4	STEMGENT, Cambridge, MA, US	Mouse IgG3	09-0006	1:200
anti-betaIII tublin (TuJ-1)	R&D systems, Minneapolis, MN, US	Mouse IgG2A	55461211	1:200
Anti-alpha-Smooth Muscle Actin	Novus, Centennial, CO, US	Mouse IgG2A	NB120-18147	1:100 - 1:200
Anti-GATA4	LifeSpanBiosciences, seattle, WA, US	Rabbit IgG	LS-C352237-100	1:100 - 1:200
Anti-GFP	MBL, Nagoya, JAPAN	Rabbit IgG	598	1:100 - 1:200
anti-HPT 2	Biorbyt, cambridge, UK	Mouse IgG	ORB383723	1:200
anti-vimentin	Thermo fisher Scientific, Waltham, MA	Mouse IgG	MA5-11883	1:200
Goat anti-Mouse IgG, Alexa Fluor 488	Thermo fisher Scientific		A11001	1:200
Goat anti-Mouse IgM Alexa Fluor 568	Thermo fisher Scientific		A21043	1:200
Goat anti-Rabbit IgG, Alexa Fluor 568	Thermo fisher Scientific		A11011	1:200
Goat anti-Mouse IgG, Alexa Fluor 568	Thermo fisher Scientific		A11004	1:200
Anti-IgG (H+L chain) (Mouse) pAb-HRP	MBL		330	1:200
Anti-IgG (H+L chain) (Rabbit) pAb-HRP	MBL		458	1:200

Supplementary Table 3 Primer sequence information for genomic PCR (Figure2a)

Species	Gene designation	F/R	Sequence	Product length (bp)
Mouse	Tsc2	Forward	TGGCATCTCCAAAACACTGCAT	640
		Reverse	TATTCCTGCGACAACCTGACC	
Chicken	Tsc2	Forward	CCGTTTGCTTGCCCTCTGATGACT	294
		Reverse	CAGCTCAGTGTTGTTCTCGGCATT	
Okinawa rail	Tsc2	Forward	TCCAGCCATCTCAGCCGCTCA	345
		Reverse	CTGCTGTTCTGCTCTTGAAGGCAT	
Japanese ptarmigan	Tsc2	Forward	CCTACCTTGTGCTGAGATGCT	653
		Reverse	TACAGACCGGCTCCATTGCTT	
Blakiston's fish owl	Tsc2	Forward	ACATCCCCACAGTTACAAAATACGC	535
		Reverse	GCTCGGGAACCCACCCTT	
Reprogramming vector	PB-TAD-7F	Forward	CTAGCGACACCGAAGAGAACGACA	432
		Reverse	CGTTTCTAGGCCACCGTTCCTGT	

Supplementary Table 4. Primer information for genomic PCR

Species	Gene name	F/R	Sequence	Product length (bp)
Japanese Golden eagle	Tsc2	Forward	CTCGCTGTTTTGTCCTCAGTGTTG	363
		Reverse	ACTATCCCCACCTTTACTATGGTC	
Reprogramming vector	PB-DDR-8F	Forward	ACTGGACCCTAGGTTCGCCAT	591
		Reverse	TCTAGGGTCCCCAGGTCAACGTTT	

Supplementary Table 5 Primer sequence information for real-time PCR (mouse)

Species	Gene name	F/R	Sequence	Product length (bp)
Mouse	Oct3/4	Forward	CCATGCATTCAAACTGAGGCACCA	132
		Reverse	ACCCCTGTTGTGCTTTTAATCCCT	
	Sox2	Forward	GCACATGGCCCAGCACTACCAG	148
		Reverse	TTGCACCCCTCCCAATTCCCT	
	Nanog	Forward	CCCTTACCCACGCCCAC	132
		Reverse	TCGAGAGTAGCCACCATATCGTT	
	Tbx3	Forward	ATTCTTGTTTTGCCTTTTGGGTCT	110
		Reverse	AGCCAGCTCTACTTGAAAGCA	
	Klf4	Forward	ATCACCTTGTATGCTCTTTGCCT	136
		Reverse	ACCTTTAGAACCACGACTCACCA	
	Klf2	Forward	ACCTGGCCTTGCACATGAAGC	177
		Reverse	AACTGGTGGCAGAGTCATTTTCCT	
	Lin28a	Forward	GACACTCCCTGGCTCTCCCAA	113
		Reverse	CCCAGCACCCCAACAAGGTTC	
	Esrrb	Forward	GTATGCTATGCCTCCCAACGA	92
		Reverse	TGAGGAACACAAGCTCCCGAT	
	Fbx15	Forward	ATGAGGACAAGAAGCCCATC	113
		Reverse	TCCTACGCTGTCCATGTACTCG	
	Prdm14	Forward	CCCATGAACCTCCGTGTCACT	100
		Reverse	ACACCTTTCCACAGCGTTCACA	
	CDH1	Forward	GCCCTCAATTACCATGTTTGCT	138
		Reverse	GCACCCACACCAAGATACCTG	
	DDX4 (VASA)	Forward	CAGTACCTATGTGCCTCCCAG	110
		Reverse	CCGCTGTATTCAACGTGTGCTT	
	Dazl	Forward	TGAGGCTCCAAATTCAGCTGT	86
		Reverse	CTTCTGGCAAAACATATCCTTGACT	
	TERT	Forward	TCATCATGTAGAGCGAGCCTT	105
		Reverse	ACAATCTGCCTTACCCATAAGTCA	
	LIF	Forward	CTTCTATACAGCCCAGCACCA	149
		Reverse	TGTGCAGCTTGTTCATACACC	
	LIFR	Forward	GATTGAGCCAAAGAACCCTCC	120
		Reverse	CCATACAACGCTGAGAAAGCC	
	FGF3	Forward	ACAGCGCCTATAGCATCCTGGAGA	119
		Reverse	ATCCGAAGCATACAGCCGTCCT	
	FGF5	Forward	TTCAAGCAGTCCGAGCAACCG	146
		Reverse	CATCCAAAGCGAAACTTCAGTCT	
	FGF8	Forward	AGCCCACTTCCCTGCTCCGA	113
		Reverse	CGTCACCAGGCTCTGCTCCCTC	
	FGF10	Forward	ATGTGCGGAGCTACAATCACC	96
		Reverse	CCTTGCCGTTCTTCTCAATCGT	
	Wnt3a	Forward	TGGCCCCTTTCCAGTCCT	148
		Reverse	AGCTTTAAAAGAGGTACCCAGGCA	
	Wnt5a	Forward	AGTTTTCTCTGGGCTTGCTCT	149
		Reverse	CCTCCGAGTCTTAGCTTGCTT	
	Gapdh	Forward	CCCCAATGTGTCCGTCGT	92
		Reverse	CCTCAGATGCCTGCTTCACC	
	c-myc	Forward	CCTCACTCAGCTCCCCTCC	105
		Reverse	AGCTTCTTTTATACTGCGACTCAGG	
	Sox3	Forward	CCCCAAGAACTGCAATGCCTCCAC	142
		Reverse	AAACACACGCACACCTGGCTA	
	Lin28b	Forward	AAATTGCGTTAGAAAGAACAAAGGA	117
		Reverse	ACATTCGGTACAAAGACAGTCC	
	Tfcp2l1	Forward	AGATCAAGGTGTTCAAGCCCAA	90
		Reverse	TCTCCTTCTCTTGAGCCGTTC	
	Sall4	Forward	CTTCACCACGAAAGGCAACCTG	144
		Reverse	GAAAACACCTCGGGCGCTCT	
	Rex1	Forward	CCCACAGCCCATCACTCCAGA	132
		Reverse	TGGACGAACAGAACTTGAGACACT	
	FGF2	Forward	AGAAGAGCGACCCACACGTCA	125
		Reverse	CTTAGAAGCCAGCAGCCGTCCA	
	Dnmt3b	Forward	AGTACCCCATCAGTTGACTTGAGC	146
		Reverse	ATCTTTCCCCACACGAGGTCAC	

Supplementary Table 6 Primer sequence information for real-time PCR (chicken)

Species	Gene name	F/R	Sequence	Product length (bp)
Chicken	Pou5	Forward	TTCCGCAAATGTGTGAAGCCCAGT	114
		Reverse	CTTGCCTTTCTGACGCCGGTT	
	Sox2	Forward	GCCGCCGCTCCGCTTC	82
		Reverse	TTTCAGCTCGGTTTCCATCATGTTGT	
	Nanog	Forward	TCTGGGGCTCACCTACAAGCA	85
		Reverse	CCACTGACTCTCTTTCTGGCAAC	
	Tbx3	Forward	CATCGCAGTGACCGCATACCAG	146
		Reverse	CGTCGTACACCCGCATGGAT	
	Klf4	Forward	CCGGACTACAAAATGCCAAGGAGT	89
		Reverse	CGTGTAATATCCCCACCCTCCGAAC	
	Klf2	Forward	AGCGATACCATCCTGCCCTCCTT	88
		Reverse	CCTCCTGCTCGCACTTCCACCT	
	Lin28a	Forward	CGGTAACAGCCACAACTCGGAA	80
		Reverse	CTTCGCACCTGCAAACTGCT	
	Esrrb	Forward	AGTCTTACTCTGCTGGGCTCT	103
		Reverse	TCATCTGGTCCAAAGCCCCTG	
	Fbx15	Forward	TGTTTCAGTGCCCCAGTTTGCAT	127
		Reverse	ACCACACCAACTCAGCACAGAC	
	Prdm14	Forward	AGAGTTTTAGCCCTGCATTTCTCG	75
		Reverse	AGAAATCAACAGAAAGCCCACACA	
	CDH1	Forward	CCTGGCAAGCCGTTTACCACA	103
		Reverse	ACCTTGGCTGTCTTCAGGATTCCG	
	DDX4 (VASA)	Forward	GACCAAACAGCCCATCCCTC	127
		Reverse	CCTTCATTAGCACCAAGTGAGC	
	Dazl	Forward	TCTGTTTAACCCTGAAAACCGTCT	86
		Reverse	TTCTGAAGTGATGCGCCCTCC	
	TERT	Forward	TTGCCAAAGTACATCTACGTGCCTT	95
		Reverse	AATGAACCGGAGCCTTGATGCAAT	
	LIF	Forward	CTCAACCGCCTCCACAACACC	119
		Reverse	TTGCTGCTCTCCCCGTAGCTC	
	LIFR	Forward	ATGGAGCAAAAGATTACCCAC	144
		Reverse	CAAACAAGACAGCTCCATCC	
	FGF3	Forward	CGCAGGAAGCTCTACTGTGCCACCA	132
		Reverse	AGCGACGATCCCGACATCAACAGCA	
	FGF5	Forward	CCCTCCTCCACCGCCACCCAA	144
		Reverse	CCACGTCCATCCCAGCGTCACC	
	FGF8	Forward	CTCTTCCATCCTCAGCGGTCT	100
		Reverse	ATCTGGGCTTCCATCAACAGG	
	FGF10	Forward	GTGCGGAGCTACAATCACCT	97
		Reverse	TGACCTTGCCGTTCTTCTCG	
	Wnt3a	Forward	AACTTTTGCGAGCCCAACCCT	93
		Reverse	AAGGTCACAACCGTCAATCCCA	
	Wnt5a	Forward	ACCTGCCCATGACTTCTGGTT	94
		Reverse	ACCCCTTATTTACAGAGCCCCAGT	
	Nestin	Forward	ATCACATCCCAAGAGCCAACCC	147
		Reverse	GCTCTGCACCTCGCTCCCA	
	Brachyury	Forward	ACCACCTCCACATCCCCACT	99
		Reverse	CCACATGGATGCCAACCGAG	
	Gata4	Forward	TTTCCCCGCAGGCTTACCAG	122
		Reverse	ACCAGTTTATGCCGTTATGATGTCC	
	Gapdh	Forward	GAGGCCAGTTCTGTTCCCTT	88
		Reverse	ATCAGTTTCTATCAGCCTCTCCC	
	c-myc	Forward	AGGAGCACTGTAAGCCCCACCAC	142
		Reverse	ACCCTGCCACTGTCCAACTTTAGCC	
	Sox3	Forward	CGCAACCGCCTCCTAAAGCC	85
		Reverse	GCGAACAAAACTACGCCCAACCC	
	Lin28b	Forward	TTTCACAACAGCCCACTAGTTCTCA	104
		Reverse	AGATGAATAGCCATACGTTCCTCCC	
	Tfcp2l1	Forward	CAAAGGAAGAAATGTAAGGCCCAA	103
		Reverse	ACTGCCATCTCCATTCTCTCG	
	Nanog-like	Forward	CAGCCAGTAGCAGTCTATCTCTGGA	148
		Reverse	AGCTGTTTTGCATGAAGCACCGAA	
	Sall4	Forward	CCCGAATCCTTTGCTACCTCAGTCC	148
		Reverse	CTCCTCTGCGCTCGATTTGCT	
	Rex1	Forward	AGGAGCCTCGTGCCCAATTCCC	142
		Reverse	CCTCTGCCTTCAAGCTCCCTGACC	
	Sox17	Forward	TGGCGCAGCAGAACCCGGACCT	72
		Reverse	ACAGCGCCTTCCACGATTTACCCA	
	Gata6	Forward	GCGTGACCCACTGCCAGCCAA	85
		Reverse	ACCGCTCCGCAGAAGAAACCC	
	Otx2	Forward	CCCTGTTCGCCAAGACCCGCTA	94
		Reverse	AACCACACCTGCACTCTGGAC	
	Pax6	Forward	AGCACAAGCGTTTACCAGCCGAT	90
		Reverse	CGCTGTGTCTGTCCTGCCCAAC	
	FGF2	Forward	ATCCGCACATCAAACTGCAGCTTC	75
		Reverse	AGCGGTTTGCACTTACGCCTT	
	Dnmt3b	Forward	GGCTCCCCTCAGTCACATCCTC	105
		Reverse	CTTTGTTTGCTTTCCCCAAGTCCT	

Supplementary Table 7 Primer sequence information for real-time PCR (Okinawa rail)

Species	Gene name	F/R	Sequence	Product length (bp)
Okinawa rail	Pou5	Forward	GCCGGACCAGCATTGAAACCAAC	79
		Reverse	TCCTGGGGACTGGGCTTCACACA	
	Sox2	Forward	ACCCCGGAGGAAAACCAAGACC	153
		Reverse	TGCGCGTAACTGTCCATCCTCT	
	Nanog	Forward	CAGAGCGCCCATCTCCCCAT	75
		Reverse	ACTTGGCCTTCTTCACCACACC	
	Tbx3	Forward	AGAAGGCCAAGTACATTTTGCTGA	93
		Reverse	CCTTGCCAGCGACCATCCAG	
	Klf4	Forward	CCCGATCCGATGAACTTACTCGT	75
		Reverse	TGTCACACCGCTGGCACT	
	Klf2	Forward	ATTAAGCCCGAGCCTGACATGGAC	91
		Reverse	TCGCGTTGCCCTCCTGT	
	Lin28a	Forward	TGAAGCCGTGGAGTTCACCTT	112
		Reverse	CTTGCCTTTGGGTCTCCTCTCG	
	Esrrb	Forward	TGGAAATCCTCATCCTGGGCAT	75
		Reverse	TGTAGTCCTCAGCATAGACGAGCTT	
	Fbx15	Forward	AAATCTGAACAAACAGAAACCGT	75
		Reverse	TTTCTTCCAGTACCCAGGCTT	
	Prdm14	Forward	AGCAGAATCTGACGGCTATCCA	118
		Reverse	CCCAGGAACTGCACGTAGCAA	
	CDH1	Forward	CGCTCAGCTCCCTCAACTCCTCC	103
		Reverse	CCGCCATAGAGGTCTGCCAGCTTC	
	DDX4 (VASA)	Forward	CAACTTCAGTGGCAGCAA	129
		Reverse	TGCCTGTATTTCCACAACGA	
	Dazl	Forward	AGTCTTCAATCCTCCTGCACCAC	103
		Reverse	ATACTGTGTTATGGGGCTCACCAC	
	TERT	Forward	TGAAGCTAACGACTCCAGCAC	133
		Reverse	TGCCTCATGTTCTGTACCCAT	
	LIF	Forward	CATCTTCTTCCCCGCTTTCCG	111
		Reverse	CGCGTGATGTTCCCCAACGA	
	LIFR	Forward	ACCCCATGATACATCTGAGCAA	104
		Reverse	ATCTTCAGACAGTATGCACACC	
	FGF3	Forward	TGCCAAACAACTACAATGCTT	88
		Reverse	TTGCCTTTCTTTGTTCTTCCA	
	FGF5	Forward	GAGCTTGCCTTCACCGTGACC	127
		Reverse	ACCCAAAGCGAAACTTCAGCCGGTA	
	FGF8	Forward	ACTGCGTCTTCACAGAGATCGTCCT	81
		Reverse	AGGCCATGTACCAGCCCTCGT	
	FGF10	Forward	TTCCCCTCGTCCTTCTCCTCT	142
		Reverse	CGCTAACCTTGCCGTTCTTCTCG	
	Wnt3a	Forward	AACTTTTGTGAGCCCAACCCT	103
		Reverse	CGCAGCACAAAAGGTCACAACCA	
	Wnt5a	Forward	TCCTGTAGCCTGAAGACCTGT	87
		Reverse	AGCAGCACTATCGTATTTCTCCT	
	Nestin	Forward	TTCCCCTGCTCCCCGGTGGCTT	100
		Reverse	TCTTGTAGGTCTCCACCGCCCCTT	
	Brachyury	Forward	ACCACCTGCTGAGCGCCGTG	121
		Reverse	AGCTCCTTGAACCGCAGCCACA	
	Gata4	Forward	TTTCCCCGCAGGCTTACCAG	86
		Reverse	AGCTAAGACCAGGCTGTTCCA	
	Gapdh	Forward	TTATCATCTCTGCCCCCTC	82
		Reverse	ATTTTCAGAGACTTGTCATACTTG	
	c-myc	Forward	TGCTTCCCTTCCACCGCCGACCA	142
		Reverse	AGCCGCTCCACATGCAGTCCT	
	Sox3	Forward	ACCTATATGAACGCCGCTTCCACCT	103
		Reverse	ATTTCACCACCGAGCCCATGGAG	
	Lin28b	Forward	ACTTCTGCTTTACCGAGGGA	136
		Reverse	ACGCAGACAACTTACTCGAC	
	Tfcp2l1	Forward	GCATCAGCACCGAATTTACTCCAC	141
		Reverse	ACACTTTGATCTGGCAGCTTG	
	Nanog-like	Forward	TGACGGCTGTAACTTCCACCAC	100
		Reverse	GCCCCACATCCAGAGTGCTT	
	Sall4	Forward	ATTGCAGCCCTGGAGAACCAA	88
		Reverse	AGCCATTGTCACTAGACTTCAAGC	
	Rex1	Forward	CAGCCTTTGCCATCCACGTT	105
		Reverse	GCGTTTCACTTTCTTCATCCCT	
	Sox17	Forward	AGCACATGCAGGACCACCCCAAC	94
		Reverse	TGCTGCAGGAAGCCGCTCTCCAC	
	Gata6	Forward	ACCAGTCCTGAAAGCAGCAAC	85
		Reverse	TCACTTCAGCCGTGGAGGTCA	
	Otx2	Forward	ACCTACACCCAGGCATCAGGCTA	149
		Reverse	ACCGCGTTGGCACCCAT	
	Pax6	Forward	CGAAACTGGCTCCATCAGACC	93
		Reverse	CGTTTATACTGCGCTATTTTGCT	
	FGF2	Forward	TCTCTACTGCAAGAACGGCGGCTT	82
		Reverse	AGGGTCGCTCTTCTCCCGGACG	
	Dnmt3b	Forward	TGAACTAGAGCGAATTATTGCTT	145
		Reverse	ATTTGGTTTCTGTATCCTGCAT	

Supplementary Table 8 Primer sequence information for real-time PCR (Japanese ptarmigan)

Species	Gene name	F/R	Sequence	Product length (bp)
Japanese Ptarmigan	Pou5	Forward	AAACCACCATCTGCCGCTTCG	103
		Reverse	TCCGTGTTCTCTGCCTCGTTG	
	Sox2	Forward	CCCCGGAGGAAAACCAAGACCCTG	100
		Reverse	CCCCTACCCCGGTCGTCATGC	
	Nanog	Forward	GCACACCAGGCTTACAGCAG	89
		Reverse	CCAAAGAACCCCTCATCCTCC	
	Tbx3	Forward	ACCGATGAGAGATCCAGTGATCCC	90
		Reverse	ACGGCGCTCATGGCAAAGTCC	
	Klf4	Forward	AACTCACTCGTCATTACCGAA	117
		Reverse	ATTAAAAGTGCCTCTTCATGTGT	
	Klf2	Forward	GGCCGCTTCTTCGTGACACC	87
		Reverse	TGGCCCATAACTGTCCATGTCAGG	
	Lin28a	Forward	CTCCCCAGCCTTGCCTT	96
		Reverse	AGCTTTCATTTGGCATTGGCTT	
	Esrrb	Forward	ACATGCTTAATGCCATCCCCAAG	89
		Reverse	TGCCTCACAGGAAGCCACACC	
	Fbx15	Forward	CTATTTCAGTTGTTTTCCAGCA	118
		Reverse	TTTCTTCCAGTATCCAGGCTT	
	Prdm14	Forward	TGTTTTCTCCTATGTAAAGATCCCC	139
		Reverse	GTGCAAGACAGCTAGTACCTT	
	CDH1	Forward	GCCTTCAGCATTATCACCGACCC	92
		Reverse	CCCACACTGGAGCCATCGACA	
	DDX4 (VASA)	Forward	TCAGTCTAGAAATTTGGGCTT	127
		Reverse	TGACTGCCGTTACTTTGGTT	
	Dazl	Forward	ATAATAAAGTAAGGCTGAGGCGTGA	100
		Reverse	CAAAATGGCCGCTCGCAGCAC	
	TERT	Forward	ACCAAATACATTCTTGCGCTCT	141
		Reverse	TCTATAAGCTGCCTTCCACCT	
	LIF	Forward	TCGACCGCCTCCACAACACC	110
		Reverse	CTCCCCATAGCTCACGTCCAC	
	LIFR	Forward	CCGCAACAACAGACACC	144
		Reverse	CCACCTGAGATAACCCCACA	
	FGF3	Forward	GCATCCCGTCTATACCGGACT	132
		Reverse	CTGTGTCCTGCGTGTTTTAAAGCC	
	FGF5	Forward	TTCAGATTGCAGCCTTCCA	91
		Reverse	ACAGCACAGAGTACCTCA	
	FGF8	Forward	GTTTGGCACCGCTCACCCAT	85
		Reverse	ACCCGCTGCCTCACCGTA	
	FGF10	Forward	CCTTAATTGCTCATTCAAGCCTA	114
		Reverse	TCCATTCTTCCACAAGGCTTC	
	Wnt3a	Forward	ACAACTTCTTCAAGGCTCCGACT	148
		Reverse	AGGTCACAGCCATCAATCCCA	
	Wnt5a	Forward	TTCCTGTAGCCTGAAGACCTG	132
		Reverse	CTGTTCATCTGCACCAGCTTG	
	Nestin	Forward	CCAACCAAGAGGAGTCCAGCATC	89
		Reverse	ATCCTTTCTGTCTTCTGCTGCTT	
	Brachyury	Forward	GTTTCTCCCACTAGCTGGCAA	102
		Reverse	CTCCAGTACATTAGGCACTTCG	
	Gata4	Forward	AAACGATAGAATTTGGCTAACTGG	142
		Reverse	ATTTCTACTATGAAAGGGCCGAGA	
	Gapdh	Forward	CCCTTTGTTGGAGCCCCTT	138
		Reverse	TTCTATCAGCCTCTCCCACCT	
	c-myc	Forward	AGGAGCACTGTAAGCCCCACCAC	142
		Reverse	ACCCTGCCACTGTCCAACTTTAGCC	
	Sox3	Forward	GCACGACCGCCTCCTAAAGCC	87
		Reverse	AGCGAACAAAACTACGCCCAACCC	
	Lin28b	Forward	GACATGAAGCTGAACCCCA	75
		Reverse	GAGATGAATAGCCATACGTTCC	
	Tfcp2l1	Forward	ATCTTCAAGCAGGAGGAACCAC	116
		Reverse	AGGGTTTCTTCGTGCAGCTTC	
	Nanog-like	Forward	AGCTTCATGACCATTGGCAAG	127
		Reverse	ATATATCTGAGTCTGGTGCCAT	
	Sall4	Forward	TCCCGAATCCTTTGCTACCTCAGT	150
		Reverse	GCTCCTCCGCATTCGATTTGCT	
	Rex1	Forward	AGCAAAGCGTTCTAAGTTGTCC	134
		Reverse	ATTCATTGAAAATCCTGAGGCACT	
	Sox17	Forward	TGGCGCAGCAGAACCCGGACCT	72
		Reverse	ACAGCGCCTTCCACGATTTACCCAG	
	Gata6	Forward	ACCTCTCTGAAAGCCGCGAGT	78
		Reverse	AATTCCCGGTGCCATCCCTCC	
	Otx2	Forward	CCCATGACCTACACCCAGGCATC	82
		Reverse	ATCCACAGTCCATCCCTCCGAA	
	Pax6	Forward	TAGCGACTCCCGAAGTTGTAAGCA	82
		Reverse	CTGTCTCGAATCTCCCACGCAAA	
	FGF2	Forward	TCAAACTCCAGCTTCAAGCAG	90
		Reverse	TGCCATCCTCCTTCATAGCC	
	Dnmt3b	Forward	ATTCACTACGTCCTTTTCCAAGCA	129
		Reverse	CAGAACATTTTCCCCTCCGGTCA	

Supplementary Table 9 Primer sequence information for real-time PCR (Blakiston’s fish owl)

Species	Gene name	F/R	Sequence	Product length (bp)
Blakiston's fish owl	Pou5	Forward	AGAAAACGCAGGACCAGCAT	78
		Reverse	ACTGGGCTTCACGCACT	
	Sox2	Forward	CTCGCTTCTCTGCTCAAGGCTGGT	121
		Reverse	TTCGAGTTGCCCGTGCCTCCC	
	Nanog	Forward	AGAGCCGCACAGCTTTCTCCA	103
		Reverse	AAAACAGCCAGCTCCCGGAT	
	Tbx3	Forward	GGTATCCCCTTTTCTTCCCTGGGTC	148
		Reverse	CCGTGCCTCTTTTATGGAATTGCT	
	Klf4	Forward	GACCACCTTGCCTTACACA	122
		Reverse	GTGTTCTCCCCATCTTGC	
	Klf2	Forward	ACTTAGCCAGTCATCTCCT	99
		Reverse	CACAGAAGCTGCATAAATACCC	
	Lin28a	Forward	CCGCGAAAGCACAGCAGTCCC	75
		Reverse	AGCTGTGCATATCCTCCTCGTCT	
	Esrrb	Forward	CGCCATCATGGAGGACTCACC	89
		Reverse	AAGCAATGTCCCCACACACC	
	Fbx15	Forward	AATGAAATTGCTTTTGTTATTGTGA	70
		Reverse	CAGAACCCAAAGTGCTTCTC	
	Prdm14	Forward	CTCTGGCAACTGGATGTCCCT	92
		Reverse	CGTAGAAGATTTGTCCCTGGCACT	
	CDH1	Forward	TGCTCCCGCCCGAGGATGACA	75
		Reverse	CCTGATCTTCCTCACCGCCACCCT	
	DDX4 (VASA)	Forward	GAGAACTCGCTCTTCACGAT	75
		Reverse	GACCTCTTGCTGCCACT	
	Dazl	Forward	AAGTGACTAATACTGACCAGC	76
		Reverse	TAAAATACAACCTCTTGGGCAAA	
	TERT	Forward	TGGAGCAAAAGCACAACCAG	82
		Reverse	TGTCTCATTTGCCAGTAGCG	
	LIF	Forward	GGCCTCATCTCCAACCTCACCT	143
		Reverse	TGCACGTACTTCCTGAGCACCT	
	LIFR	Forward	GACTCAGAAACGGAGAACCAC	105
		Reverse	CACCTGAGATGACCCCACA	
	FGF3	Forward	CGCAGGAAACTCTACTGTGCCACCA	84
		Reverse	GCTGTTTTTCTCCAGGGTGCCGTTG	
	FGF5	Forward	ACACCTATGCCTCAGCTGTCCACC	76
		Reverse	TTGCCCCTCTTGTTGAGTGCCACA	
	FGF8	Forward	ACTGTGTCTTCACGGAGATCGTCCT	82
		Reverse	AAGGCCATGTACCAGCCCTCG	
	FGF10	Forward	ATTAATATGCCCATTCAGCAC	85
		Reverse	TCCTTATCTAGGTATCGCTCT	
	Wnt3a	Forward	AACTTTTGTGAGCCCAACCCT	92
		Reverse	AGGTCGCAACCATCAATCCCA	
	Wnt5a	Forward	TTCAGTACATTTAGGGCTCTGCAA	117
		Reverse	GCTCTTTACCCTCCGATGACA	
	Nestin	Forward	AGCAAGCAGCCCGAGAAGAAGCCAA	139
		Reverse	TGCACCTCCAGCAAGGCTTCCAC	
	Brachyury	Forward	ATCCCTCAATCTACGGTGTACTCT	80
		Reverse	ACATCCATGCTGTTCAATGTGCC	
	Gata4	Forward	CACCGTCTTCTCACTACCCAA	126
		Reverse	GCCCTAGACCATATCAAATCCAC	
	Gapdh	Forward	CCTTTTCACCACCGCTTAGCTCT	114
		Reverse	CTATCAGCCTCTCCACCTCCC	
	c-myc	Forward	AGCTTCATCTGCGACCCGGACGAC	73
		Reverse	AGCCGCTCCACATGCAGTCCT	
	Sox3	Forward	TCACAGACCCTTCCTCCCTGC	89
		Reverse	TACGGTACCATTGACGCCAGT	
	Lin28b	Forward	GCGAAAGAATGTAGTCTACCTCC	135
		Reverse	TTCAGCTTCGTGTCTTCCC	
	Tfcp2l1	Forward	AACTTGTACAGCATTTCTCCAC	80
		Reverse	TTGCTCACTAATACATGGATCCCT	
	Nanog-like	Forward	AACATCCAAACTGTGCCTCCC	87
		Reverse	GACTCCTCCATCTTCGCTGGT	
	Sall4	Forward	CCGTCATCTTCCAAAATCCGCTT	104
		Reverse	CGGTTTGCTCTCAGACACGCTA	
	Rex1	Forward	ACCCACTTTCAAATTACTCAGCC	89
		Reverse	ATAACTAGACACCCTACCCCTT	
	Sox17	Forward	GCAGCAAGAGCGAGGCGCGGAT	70
		Reverse	TTGCGCTCGTCCTTCGCCCACACC	
	Gata6	Forward	CACCAGTCCCGAAAGCAGCAA	102
		Reverse	TGTCTCACAGATGCCGTCACT	
	Otx2	Forward	GCCCTTTTCGCCAAAACCCGCTAC	70
		Reverse	GCAGGTTGATTTTCAGCGCCACCTC	
	Pax6	Forward	TAGCGACTCCAGAAGTTGTAAGCA	116
		Reverse	ATTATCGTTGGTACAGACCCC	
	FGF2	Forward	AGTCAAACTGCAACTTCAAGC	92
		Reverse	TGCCATCCTCCTTCATAGCC	
	Dnmt3b	Forward	GCATGAAATACTCCGTTTTAGCC	108
		Reverse	ATTCGCTATAGTTCAAGTTTGCC	

Supplementary Table 10. Primer information for qPCR

Species	Gene name	F/R	Sequence	Product length (bp)
Japanese Golden Eagle	Sox3 like	Forward	ACAACTCCTGCCTCCTAGAAGCC	93
		Reverse	AGCGAATAAAACAGTGCCCAACC	
	Nanog like	Forward	AGCAGAAACCTTCAGGCTGTGACC	107
		Reverse	CCCTCATCCTCCACAGCCACGAA	
	Lin28a	Forward	CGCCCGTCGATGTCTTCGTACACC	81
		Reverse	ACTCAACAGCTTCGCCCTCCTT	
	Lin28b	Forward	AGGAATCCCAGGTTTTGCAC	75
		Reverse	TGGAGATGAACCCGAATCCC	
	Sox2	Forward	CCCGGAGGAAAACCAAGACCCTCA	103
		Reverse	CCAACCCCTACCCCGGTCGTC	
	Tert	Forward	AGCAACAACTTACGCGCTCCTC	100
		Reverse	GCTCCCCAAGGGCTGAATCACAC	
	Cdh1	Forward	CACCGACCCCAAAACCAACGA	102
		Reverse	ACAGCGTTCTCCACCGTTACCAG	
	Sall4	Forward	TGCCAGAAAACACCTGCGACA	145
		Reverse	TTTGAAGAACTCCTAGGGCCAT	
	Esrrb	Forward	ACCAAAATGAAATGCACACCAC	102
		Reverse	CTTAGGATCTTTTCTGCCCAT	
	Fbxo15	Forward	ATACCATCTTGCTAACGTGAC	90
		Reverse	ACTAAGAGTCCTGGCTTCAGA	
	Gapdh	Forward	ACCATCTTCCAGGAGCGTGACCC	134
		Reverse	ACACGCTTAGCACCACCCTT	
	Nestin	Forward	AAGCAACCGAAGCAGGCAAA	83
		Reverse	TAAATGCCTCAAAGTGCCCTTCGT	
	Pax6	Forward	GGACCCACTATCCCGATGTGT	141
		Reverse	CTTGTCTCCGCTGGTTCCTCA	
	Otx2	Forward	ACGCTCCAGTTTTAGTCAGGT	124
		Reverse	AGTCTGAACGGGATCACACCA	
	Brachyury	Forward	CAGCACCGGCACAGCTACCAG	101
		Reverse	CGTTGGACATCCCGCTCGACT	
	Gata4	Forward	GCCACCATCTTCTCATTACCCAA	114
		Reverse	CAAATCCGCTAAAGCCACCAC	
	Gata6	Forward	ACCAGTCCCGAAAGCAGCAAC	103
		Reverse	CCTGTCTCACCGATGCCGTCA	

Supplementary Table 11 Primer sequence information for genomic PCR (Figure 8f)

Species	Gene name	F/R	Sequence	Product length (bp)
Chicken	Tsc2	Forward	CCGTTTGCTTGCCCTCTGATGACT	294
		Reverse	CAGCTCAGTGTTGTTCTCGGCATT	
Reprogramming vector	PB-TAD-7F	Forward	CAGCCCCAGTCGAGTACCCAT	250
		Reverse	TCCTCCCCGAAGTTGTGCAGT	

Supplementary Table 12 Primer sequence information for real-time PCR (Figure 8g, h)

Species	Gene name	F/R	Sequence	Product length (bp)
Chicken	Tsc2	Forward	TCTGTAAGCCCAAAGAATAAGCA	117
		Reverse	AGCTTATCACCCACACCA	
		probe	[FAM]CACACAGCACCTTAGCTAGAGCAC[BHQ1]	
Reprogramming vector	PB-TAD-7F	Forward	GACAACAATTTGCCGGTTCGAGG	91
		Reverse	GGCTTCTTCCACCCACTTCTCCA	
		probe	[FAM]ACATGTGCAAGCTGAGGCCACT[BHQ1]	

Supplementary references

1. Katayama M*, et al.* Induced pluripotent stem cells with six reprogramming factors from Prairie Vole, which is an animal model for social behaviors. *Cell Transplant* **25**, 783-796 (2016).

2. Katayama M*, et al.* Immortalized prairie vole-derived fibroblasts (VMF-K4DTs) can be transformed into pluripotent stem cells and provide a useful tool with which to determine optimal reprogramming conditions. *J Reprod Dev* **63**, 311-318 (2017).
