# Supplementary material for: Induced pluripotent stem cells of endangered avian species
Source: Commun Biol. 2022 Oct 24;5:1049. doi: 10.1038/s42003-022-03964-y (PMC9592614; doi:10.1038/s42003-022-03964-y)
Supplement: Supplementary file 3 — Description of Additional Supplementary Files [file 42003_2022_3964_MOESM3_ESM.pdf]

Description of Additional Supplementary Files

File name: Supplementary Data
Description: All source data underlying the graphs presented in the main figures.
