# Supplementary material for: Induced pluripotent stem cells of endangered avian species
Source: Commun Biol. 2022 Oct 24;5:1049. doi: 10.1038/s42003-022-03964-y (PMC9592614; doi:10.1038/s42003-022-03964-y)
Supplement: Supplementary file 5 — Reporting Summary [file 42003_2022_3964_MOESM5_ESM.pdf]

nature portfolio

Corresponding author(s): DBPR, COMMSBIO-20-3617B

Last updated by author(s): Jul 13, 2022

Reporting Summary

Nature Portfolio wishes to improve the reproducibility of the work that we publish. This form provides structure for consistency and transparency in reporting. For further information on Nature Portfolio policies, see our Editorial Policies and the Editorial Policy Checklist.

Statistics

For all statistical analyses, confirm that the following items are present in the figure legend, table legend, main text, or Methods section.

n/a	Confirmed	
☐	☒	The exact sample size (*n*) for each experimental group/condition, given as a discrete number and unit of measurement
☐	☒	A statement on whether measurements were taken from distinct samples or whether the same sample was measured repeatedly
☐	☒	The statistical test(s) used AND whether they are one- or two-sided<br>*Only common tests should be described solely by name; describe more complex techniques in the Methods section.*
☐	☒	A description of all covariates tested
☐	☒	A description of any assumptions or corrections, such as tests of normality and adjustment for multiple comparisons
☐	☒	A full description of the statistical parameters including central tendency (e.g. means) or other basic estimates (e.g. regression coefficient) AND variation (e.g. standard deviation) or associated estimates of uncertainty (e.g. confidence intervals)
☒	☐	For null hypothesis testing, the test statistic (e.g. *F*, *t*, *r*) with confidence intervals, effect sizes, degrees of freedom and *P* value noted<br>*Give P values as exact values whenever suitable.*
☒	☐	For Bayesian analysis, information on the choice of priors and Markov chain Monte Carlo settings
☒	☐	For hierarchical and complex designs, identification of the appropriate level for tests and full reporting of outcomes
☒	☐	Estimates of effect sizes (e.g. Cohen's *d*, Pearson's *r*), indicating how they were calculated

Our web collection on statistics for biologists contains articles on many of the points above.

Software and code

Policy information about availability of computer code

Data collection	No software was used.
Data analysis	CLC workbench<br>build number, 20210816012301

For manuscripts utilizing custom algorithms or software that are central to the research but not yet described in published literature, software must be made available to editors and reviewers. We strongly encourage code deposition in a community repository (e.g. GitHub). See the Nature Portfolio guidelines for submitting code & software for further information.

Data

Policy information about availability of data

All manuscripts must include a data availability statement. This statement should provide the following information, where applicable:

- Accession codes, unique identifiers, or web links for publicly available datasets
- A description of any restrictions on data availability
- For clinical datasets or third party data, please ensure that the statement adheres to our policy

We have provided a full data availability statement in the manuscript.

Human research participants

Policy information about studies involving human research participants and Sex and Gender in Research.

Reporting on sex and gender	Not applicable.
Population characteristics	Not applicable.
Recruitment	Not applicable.
Ethics oversight	Not applicable.

Note that full information on the approval of the study protocol must also be provided in the manuscript.

Field-specific reporting

Please select the one below that is the best fit for your research. If you are not sure, read the appropriate sections before making your selection.

☒ Life sciences ☐ Behavioural & social sciences ☐ Ecological, evolutionary & environmental sciences

For a reference copy of the document with all sections, see nature.com/documents/nr-reporting-summary-flat.pdf

Life sciences study design

All studies must disclose on these points even when the disclosure is negative.

Sample size	No sample-size calculation was performed.
Data exclusions	No data were excluded from the analyses.
Replication	All attempts at replication were successful, confirm this.
Randomization	Allocation was random.
Blinding	Blinding was possible.

Reporting for specific materials, systems and methods

We require information from authors about some types of materials, experimental systems and methods used in many studies. Here, indicate whether each material, system or method listed is relevant to your study. If you are not sure if a list item applies to your research, read the appropriate section before selecting a response.

Materials & experimental systems

n/a	Involved in the study	
☐	☒	Antibodies
☒	☐	Eukaryotic cell lines
☒	☐	Palaeontology and archaeology
☐	☒	Animals and other organisms
☒	☐	Clinical data
☒	☐	Dual use research of concern

Methods

n/a	Involved in the study	
☒	☐	ChIP-seq
☐	☒	Flow cytometry
☒	☐	MRI-based neuroimaging

Antibodies

Antibodies used

SSEA-1; STEMGENT, Mouse IgM, 09-0005, 2438.
SSEA-3; Bioss, Rabbit IgG, bs-3575R, Af02159489.
SSEA-4; STEMGENT, Mouse IgG3, 09-0006, J15110000000022.
anti-betaIII tublin (TuJ-1); R&D systems, Mouse IgG2A, 55461211, HGQ0116111.
Anti-alpha-Smooth Muscle Actin; Novus, Mouse IgG2A, NB120-18147, L14012866.
Anti-GATA4; LifeSpanBiosciences, Rabbit IgG, LS-C352237-100, 105019.
anti-HPT 2; Biorbyt, Mouse IgG, ORB383723, AB7171.
Goat anti-Mouse IgG Alexa Fluor 488; Thermo fisher, A11001, 2051236.

	Goat anti-Mouse IgM Alexa Fluor 568; Thermo fisher, A21043, 1786285.<br>Goat anti-Rabbit IgG Alexa Fluor 568; Thermo fisher, A11011, 1842719.<br>Goat anti-Mouse IgG Alexa Fluor 568; Thermo fisher, A11004, 1793903.<br>Anti-GFP MBL, Rabbit IgG, 598<br>anti-vimentin Thermo Fisher Scientific, Mouse IgG, MA5-11883<br>Anti-IgG (H+L chain) (Mouse) pAb-HRP, MBL, 330<br>Anti-IgG (H+L chain) (Rabbit) pAb-HRP, MBL, 458
Validation	Data provided in the manuscript.

Animals and other research organisms

Policy information about studies involving animals; ARRIVE guidelines recommended for reporting animal research, and Sex and Gender in Research

Laboratory animals	SCID mouse, C.B-17/Icr-scid/scidJcl; 5 to 6 weeks.
Wild animals	Somatic cells were obtained from wild animals (ex., Okinawa rail). The sampling details described below do not include the exact location of sampling to protect against poaching.<br>Fibroblast cells from Okinawa rail and Japanese ptarmigan were obtained from dead animals, such as those killed by vehicles. Approval was not required to obtain these samples.<br>Dead Okinawa rail were found on May 21, 2008, by the Okinawa Wildlife Federation, a nonprofit organization that focuses on the conservation of wild animals in the Okinawa area in the southwest region of Japan. The organization has permission from the Japanese Ministry of the Environment (MOE) to handle and perform first aid activities on endangered animals. The dead birds were transferred the following day to the National Institute for Environmental Studies (NIES). Primary cell culture was carried out from muscle tissue and skin of the dead birds (NIES ID: 715A).<br>On July 8, 2004, tissues recovered from dead Japanese ptarmigan (e.g., skin and retina tissues) were also transferred to NIES from Gifu University Department of Veterinary Medicine. Primary cell culture from this tissue was performed (NIES ID: 22A).
Reporting on sex	Not applicable.
Field-collected samples	Somatic cells from Blakiston's fish owl and Japanese golden eagle were obtained from emerging pinfeathers. Concerning the Blakiston's fish owl, the MOE carries out bird banding, of wild birds with identification tags. The emerging pinfeathers we used had been accidentally release during banding. The banding had been performed by a veterinarian at the Institute for Raptor Biomedicine Japan (IRBJ) in the Hokkaido area on June 2, 2006. IRBJ is a private organization that primarily focuses on emergency medicine first aid and care for wild avians in Hokkaido region of Japan. IRBJ is contracted to MOE to handle and administer first aid for endangered animals. The MOE banding ring was 14C0242. Since banding was carried out with the permission of MOE for capturing wildlife, we did not require the approval to obtain these avian somatic cells. On July 8, 2006, Blakiston's fish owl pinfeathers were transferred to from IRBJ to NIES, where primary cell culture was performed (NIES ID: 215A).<br>Concerning the Japanese golden eagle, an emerging pinfeather accidentally fell off a bird during blood collection at the Yagiyama Zoo in Sendai, Japan on July 11, 2018. Dr. Yukiko Watanabe, an IRBJ veterinarian, collected the emerging pinfeather. The sample was shipped the following day to NIES where primary cell culture was performed (NIES ID: 5228).<br>In addition to these birds, we obtained somatic cells emerging avian pinfeathers of Steller's sea eagle, white-tail eagle, mountain hawk-eagle, northern goshawk, Taiga bean goose, and Latham's snipe. These samples were provided by IRBJ.<br>Concerning the Steller's sea eagle, an injured individual was found in Hokkaido on July 11, 2006 (ID: 06-NE-SSE-1). The eagle was transferred to IRBJ. On December 4, 2006, IRBJ veterinarian Dr. Keisuke Saito collected fallen pinfeathers. Primary cell culture was performed at NIES on December 8, 2006 (NIES ID: 369A).<br>Concerning the white-tailed eagle, an injured individual was found in Hokkaido, Japan, on July 12, 2007 (ID: 07-NE-WTE-4). The bird was transferred to IRBJ the same day for emergency treatment. On January 15, 2008, Dr. Saito collected fallen pinfeathers. Primary cell culture was performed on January 18, 2008 at NIES (NIES ID: 492A).<br>Concerning the mountain hawk-eagle, an injured individual was found in the Hokkaido area on August 10, 2008 (ID: 08-Tokachi-HHE-2). The bird was transferred to IRBJ the same day. The bird was treated by an IRBJ veterinarian, but died on September 8, 2008. Emerging pinfeathers were collected from the dead bird by Dr. Saito. Primary cell culture was performed on September 11, 2008 at NIES (NIES ID: 847A).<br>Concerning the Northern Goshawk, IRBJ accepted an injured bird for treatment on June 12, 2006. Following treatment and recovery, the bird was released into the wild in the Hokkaido area on August 1, 2006. During the treatment (July 4, 2006), Dr. Saito collected fallen pinfeathers. The primary cell culture was performed at NIES on July 6, 2006 (NIES ID: 222A).<br>Concerning the Taiga bean geese, an injured individual was found in Hokkaido on September 15, 2016 (ID: 13B8005). The injured bird was transferred to IRBJ the same day for emergency treatment. On September 16, 2016, IRBJ veterinarian Dr. Yukiko Watanabe collected fallen emerging pinfeathers. Primary cell culture was performed on September 20, 2016 (NIES ID: 4420A).<br>Finally, concerning the Latham's snipe, fallen pinfeathers were collected during MOE approved bird banding performed on September 17, 2006, by Dr. Saito. Dr. Saito also collected fallen emerging pinfeathers (ID: 6A22598). The samples were transferred to NIES on September 20, 2006, for primary cell culture (NIES ID: 338A).<br>All records are available at NIES.
Ethics oversight	In this study, we carried out teratoma formation experiments at Iwate University. Therefore, all surgical procedures and animal husbandry were carried out in accordance with international guidelines with the Animal Experiments of Iwate University, and were approved by the Animal research committee of Iwate University (approved number A201734).

Note that full information on the approval of the study protocol must also be provided in the manuscript.

Flow Cytometry

Plots

Confirm that:

☒ The axis labels state the marker and fluorochrome used (e.g. CD4-FITC).

☒ The axis scales are clearly visible. Include numbers along axes only for bottom left plot of group (a 'group' is an analysis of identical markers).

☒ All plots are contour plots with outliers or pseudocolor plots.

☒ A numerical value for number of cells or percentage (with statistics) is provided.

Methodology

Sample preparation	According to manufacture protocol (Muse Cell Cycle Assay Kit), we preprepared the samples.
Instrument	Muse cell cycle analyzer (Luminex Corporation, 0500-3115)
Software	Built-in software of Muse Cell Analyzer
Cell population abundance	We did not use the flow cytometer as cell sorter.
Gating strategy	According to manufacture protocol (Muse Cell Cycle Assay Kit), we defined the cell populations.

☐ Tick this box to confirm that a figure exemplifying the gating strategy is provided in the Supplementary Information.
